# Distinct cell type-specific protein signatures in *GRN* and *MAPT* genetic subtypes of frontotemporal dementia

**DOI:** 10.1186/s40478-022-01387-8

**Published:** 2022-07-07

**Authors:** Suzanne S. M. Miedema, Merel O. Mol, Frank T. W. Koopmans, David C. Hondius, Pim van Nierop, Kevin Menden, Christina F. de Veij Mestdagh, Jeroen van Rooij, Andrea B. Ganz, Iryna Paliukhovich, Shamiram Melhem, Ka Wan Li, Henne Holstege, Patrizia Rizzu, Ronald E. van Kesteren, John C. van Swieten, Peter Heutink, August B. Smit

**Affiliations:** 1 Department of Molecular and Cellular Neurobiology, Center for Neurogenomics and Cognitive Research, Amsterdam Neuroscience, Vrije Universiteit Amsterdam, W&N Building, C314. De Boelelaan 1105, 1081 HV Amsterdam, The Netherlands; 2 Department of Neurology, Erasmus Medical Center, Rotterdam, The Netherlands; 3 German Center for Neurodegenerative Diseases (DZNE)-Tübingen, Tübingen, Germany; 4 Department of Clinical Pharmacy and Pharmacology, University Medical Center Groningen, Groningen, the Netherlands; 5 Alzheimer Center, Department of Neurology, Amsterdam Neuroscience, VU University Medical Center, Amsterdam, The Netherlands; 6 Department of Internal Medicine, Erasmus Medical Center, Rotterdam, The Netherlands; 7 Department of Clinical Genetics, Amsterdam Neuroscience, VU University Medical Center, Amsterdam, The Netherlands; 8 Department of Clinical Genetics, Erasmus Medical Center, Rotterdam, The Netherlands

**Keywords:** Frontotemporal dementia, *GRN*, *MAPT*, Human brain proteomics, Cell type enrichment

## Abstract

Frontotemporal dementia is characterized by progressive atrophy of frontal and/or temporal cortices at an early age of onset. The disorder shows considerable clinical, pathological, and genetic heterogeneity. Here we investigated the proteomic signatures of frontal and temporal cortex from brains with frontotemporal dementia due to *GRN* and *MAPT* mutations to identify the key cell types and molecular pathways in their pathophysiology. We compared patients with mutations in the *GRN* gene (n = 9) or with mutations in the *MAPT* gene (n = 13) with non-demented controls (n = 11). Using quantitative proteomic analysis on laser-dissected tissues we identified brain region-specific protein signatures for both genetic subtypes. Using published single cell RNA expression data resources we deduced the involvement of major brain cell types in driving these different protein signatures. Subsequent gene ontology analysis identified distinct genetic subtype- and cell type-specific biological processes. For the *GRN* subtype, we observed a distinct role for immune processes related to endothelial cells and for mitochondrial dysregulation in neurons. For the *MAPT* subtype, we observed distinct involvement of dysregulated RNA processing, oligodendrocyte dysfunction, and axonal impairments. Comparison with an in-house protein signature of Alzheimer’s disease brains indicated that the observed alterations in RNA processing and oligodendrocyte function are distinct for the frontotemporal dementia *MAPT* subtype. Taken together, our results indicate the involvement of different brain cell types and biological mechanisms in genetic subtypes of frontotemporal dementia. Furthermore, we demonstrate that comparison of proteomic profiles of different disease entities can separate general neurodegenerative processes from disease-specific pathways, which may aid the development of disease subtype-specific treatment strategies.

**Supplementary Information:**

The online version contains supplementary material available at 10.1186/s40478-022-01387-8.

## Background

Frontotemporal dementia (FTD) refers to a spectrum of neurological disorders characterized by progressive atrophy of frontal and/or temporal cortices, with an early age of onset. FTD displays heterogeneity in clinical symptoms, pathological hallmarks, and genetic aetiology. Up to 30% of patients present with a genetic autosomal dominant inheritance pattern, in majority evoked by a repeat expansion in the *C9ORF72* gene (FTD-C9), or mutations in the progranulin gene (FTD-GRN) or the microtubule-associated protein tau gene (FTD-MAPT) [27, 61, 64]. The neuropathological hallmark of FTD is specific proteinopathy, with 50% of the cases showing TDP-43 aggregates, 40% tau aggregates, and 5 ~ 10% showing FET protein family aggregates [5, 42].

While TDP-43 aggregates are classically linked to FTD-C9 and FTD-GRN, and tau- aggregates to FTD-MAPT [5], neuropathological features transcend specific subtypes, and combinations of neuropathological and clinical features are seen in sporadic FTD. This heterogeneity, combined with a relatively rare occurrence (3–26 in 100.000 people worldwide [71]), makes it challenging to study the disease in humans on a large scale and in a stratified manner. Cell and animal models of FTD have revealed potential disease mechanisms, however, demonstrating their involvement in distinct subtypes has proven difficult and attempts to translate findings into therapeutic strategies have failed so far [2, 23, 60].

Key to the development of treatment strategies is to identify cell type-specific pathways that drive disease initiation and progression. To date, several genome-wide association and transcriptomic studies have identified susceptibility genes, implicating impairments in lysosomal autophagy and the immune system across the FTD spectrum [6, 8, 18, 19, 63, 68]. A few studies have focussed on proteome changes in neuropathological subtypes, including FTD associated with TDP-43 pathology (FTD-TDP) [25, 26, 31, 38, 48, 70], FUS pathology [43], and in the genetic subtype FTD-C9 [3]. However, a systematic proteomic analysis of dysregulated proteins and pathways in affected cell types in genetic FTD is lacking.

Here, we performed a stratified analysis of two genetic subtypes, FTD-GRN and FTD-MAPT, to enable identification of disease mechanisms that are either shared or distinct for these subtypes. Data-independent quantitative proteomic analysis (DIA) of frontal and temporal cortical tissues from FTD patients with genetically-confirmed *GRN* or *MAPT* mutations, and non-demented controls (NDCs) was performed. Brain region-specific protein expression profiles for both subtypes were identified. Expression-weighted cell type enrichment (EWCE) analysis was performed to reveal cell types contributing to the FTD subtype-specific disease processes. Subsequent gene ontology (GO) analysis uncovered biological processes that are distinct for the different FTD subtypes and the cell types involved. Finally, by comparing FTD-MAPT with Alzheimer’s disease (AD), both general neurodegenerative and FTD-specific processes were revealed.

## Materials & methods

### Selection of FTD cases

**Fig. 1:**
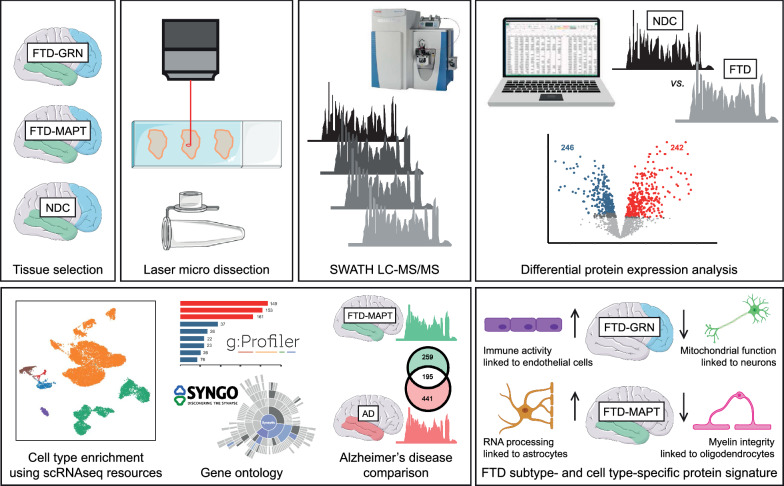
RiMOD-FTD study workflow. After tissue preparation of all 66 brain cortical tissue samples, we performed DIA mass spectrometry (SWATH), followed by differential protein expression analysis. The identified proteins with differential expression in the FTD subtypes were further subjected to downstream bioinformatics analyses. These included (1) expression-weighted cell type enrichment based on existing single cell RNAseq (scRNAseq) data resources, (2) several gene ontology analyses, and (3) an extensive comparison with an in-house sporadic AD proteomic data set. The results of these analyses were integrated and led to the identification of distinct cell type-specific protein signatures in GRN and MAPT genetic FTD subtypes

A schematic overview of the workflow is presented in Fig. 1. Post-mortem brain tissues were obtained from the Netherlands Brain Bank, Netherlands Institute for Neuroscience, Amsterdam, and from the Queen Square Brain Bank for Neurological Disorders, UCL Institute of Neurology, London. All materials have been collected from donors from whom written informed consent for brain autopsy and the use of the material and clinical information for research purposes has been obtained. Approval was granted for the Netherlands Brain Bank by the Ethics Committee of the Vrije Universiteit Medical Center (April 30, 2009), and for the Queen Square Brain Bank for Neurological Disorders by the London Central Research Ethics Committee (August 6, 2013). A total of 22 brains from patients with familial FTD were available and eligible based on clinical and neuropathological reports, and genetic origin was validated by genetic screening. Cortical tissues from the middle frontal gyrus and middle temporal gyrus were obtained from nine cases with FTD-GRN and 13 cases with FTD-MAPT. In addition, middle frontal and temporal cortical tissues were obtained from 11 sex-matched non-demented controls. Extended information on all cases and controls in the RiMOD-FTD cohort is listed in Table 1.

**Table 1:** Demographic, clinical, and post-mortem characteristics for cortical frontal and temporal samples from the RiMOD-FTD cohort

	NDC (N = 11)	FTD-GRN (N = 9)	FTD-MAPT (N = 13)
*Demographics*			
Female, n (%)	F: 7/11 (63,6)	F: 5/8 (62,5)	F: 5/11 (45,5)
	T: 5/8 (62,5)	T: 5/9 (55,6)	T: 5/13 (38,5)
Age, median (range)	F: 83 (60–91)	F: 65 (52–76) ‡	F: 60 (49–75) ‡
	T: 83 (60–91)	T: 65 (52–76) †	T: 60 (46–75) ‡
*Clinical characteristics*			
Genetic mutation (n)	n/a	C105fs (1)	G272V (4)
		G24X (1)	P301L (8)
		G125X (1)	R406V (1)
		G300X (1)	
		S82VfsX174 (4)	
		C31LfsTer34 (1)	
Disease pathology (n)	NA	F | T:	F | T:
• Atypical Pick’s disease		– | –	4 | 4
• Tau		– | –	7 | 9
• TDP-type A		8 | 8	– | –
• Undetermined		– | 1	– | –
ApoE profile (n)	F | T:	F | T:	F | T:
• 32	– | –	2 | 2	1 | 1
• 33	6 | 3	3 | 3	5 | 7
• 42	– | –	– | –	– | –
• 43	2 | 2	– | –	3 | 3
• 44	1 | 1	– | –	1 | 1
• Unknown	2 | 2	3 | 4	1 | 1
*Post-mortem determinants*			
Post-mortem delay, median (range)	F: 05:50 (03:35–08:00)	F: 05:00 (03:35–06:05) *	F: 05:23 (04:10–11:30)
	T: 05:54 (03:35–08:00)	T: 05:00 (03:35–06:05)	T: 05:35 (04:10–11:30)
Cerebrospinal fluid pH, median (range)	F: 6.75 (6.26–7.20)	F: 6.36 (6.10–6.52) †	F: 6.46 (5.91–6.64) †
	T: 6.72 (6.26–7.20)	T: 6.36 (6.10–6.52) †	T: 6.46 (5.91–6.64) *
Brain weight in grams, median (range)	F: 1188 (943–1590)	F: 954 (830–1242) *	F: 962 (652–1188) †
	T: 1130 (943–1590)	T: 954 (830–1242) *	T: 1011 (652–1188) *
Braak score, median (range)	F: 1 (0–2)	n/a	n/a
	T: 2 (0–2)		

Expected ‘healthy control’ donors that enter the Netherlands Brain Bank are assessed by a neuropathologist for the presence of neurodegenerative pathology with an extensive (immuno)histochemical assessment. A Braak stage score < IV in combination with the absence of any clinical signs of dementia is handled to assign them the label of non-demented control (NDC). * Significant difference compared with NDC data (Student’s t-test; *p* < 0.05), † Significant difference compared with NDC data (Student’s t-test; *p* < 0.01), ‡ Significant difference compared with NDC data (Student’s t-test; *p* < 0.001). F; cortical frontal tissue, T; cortical temporal tissue, NA; not applicable, n/a; not available.

### Immunohistochemistry for neuropathological characterization

Routine immunohistochemistry was carried out by the Netherlands Brain Bank, Netherlands Institute for Neuroscience, Amsterdam. In addition, we performed extended staining on multiple brain regions, including all cortical areas, hippocampus, caudate nucleus, and putamen, using AT8 (MN1020, Thermo Fisher Scientific, 1:400) and pTDP-43 (CAC-TIP-PTD-M01, Cosmo Bio, 1:1000) antibodies. The pattern of TDP-43 pathology was classified according to the morphology and distribution of neuronal inclusions as proposed by Neumann et al. [50].

### Brain tissue preparation and laser microdissection

Sections (10 µm) of fresh frozen tissue were mounted on polyethylene naphthalate-membrane slides (Leica, Herborn, DE), fixed in 100% ethanol for 1 min and stained using 1% (wt/vol) Toluidine Blue in H_2_O (Fluka Analytical, Buchs, Switzerland) for 1 min. Laser microdissection was performed using a Leica AS LMD system. A volume of 1.2 mm^3^ of grey matter tissue from the frontal and temporal cortical regions was collected in Eppendorf tubes containing 30-µL M-PER lysis buffer (Thermo Scientific, Rockford, IL, USA) supplemented with reducing sodium dodecyl sulphate sample buffer (Thermo Scientific). Microdissected tissue was stored at −80 °C until further use.

### Protein separation by electrophoresis and in-gel digestion

Microdissected tissue lysates were incubated at 95 °C for 5 min, followed by incubation with 50-mM iodoacetamide for 30 min at room temperature in the dark. Proteins were size separated on a NuPAGE 4–12% Bis–Tris acrylamide gel (Invitrogen, Carlsbad, CA, USA) using MOPS sodium dodecyl sulphate running buffer (Invitrogen) according to the manufacturer’s protocol. Gels were fixed and stained with colloidal Coomassie Blue G-250 overnight while shaking. After destaining in ultrapure H_2_O, each gel lane was sliced into four equal-sized parts and each part was cut into blocks of approximately 1 mm^3^ and collected in a 96-wells plate. Destaining, trypsin digestion, and peptide extraction were done as described previously [11].

### Micro LC and data-dependent acquisition mass spectrometry of strong cation-exchange fractions for library preparation

For library preparation, pooled protein extracts from a mix of FTD-MAPT and NDC samples were used. Extracted peptides were analysed by micro liquid chromatography with tandem mass spectrometry (LC–MS/MS) using an Ultimate 3000 LC system (Dionex, Thermo Scientific) coupled to the TripleTOF 5600 mass spectrometer (Sciex). Peptides were trapped on a 5 mm Pepmap 100 C18 column (300 μm i.d., 5 μm particle size, Dionex) and fractionated on a 200 mm Alltima C18 column (300 μm i.d., 3 μm particle size). The acetonitrile concentration in the mobile phase was increased from 5 to 18% in 88 min, to 25% at 98 min, 40% at 108 min and to 90% in 2 min, at a flow rate of 5 μL/min. The eluted peptides were electro-sprayed into the TripleTOF MS with a micro-spray needle voltage of 5,500 V. The mass spectrometer was operated in a data-dependent acquisition (DDA) mode with a single MS full scan (m/z 350–1250, 150 ms) followed by a top 25 MS/MS (m/z 200–1800, 150 ms) at high sensitivity mode in UNIT resolution, precursor ion > 150 counts/s, charge state from + 2 to + 5, with an exclusion time of 16 s once the peptide was fragmented. Ions were fragmented in the collision cell using rolling collision energy, and a spread energy of 5 eV.

### Micro LC and data-independent acquisition mass spectrometry for experimental samples

The conditions used for micro liquid chromatography of experimental samples were the same as those for the library preparation. The mass spectrometer was operated in a data-independent acquisition (DIA) mode, where experiments consisted of a parent ion scan of 150 ms followed by a window of 8 Da with scan time of 80 ms, and stepped through the mass range between 450 and 770 m/z. The total cycle time was about 3.2 s, which yielded in general 9–10 measurement points across a typical peptide with an elution time of 30 s. The collision energy for each window was determined based on the appropriate collision energy for a 2 + ion, centered upon the window with a spread of 15 eV.

### DIA data extraction and analysis

We first analysed the DDA data from our pooled library samples and identified 3,422 protein groups by MaxQuant search (version 1.5.2.8) [13] against the human proteome using the UniProt FASTA (release February 2015) and Biognosys iRT FASTA databases. The proteins and their corresponding fragment ions were then converted into a spectral library with Spectronaut, version 11 [9], for which the Q-value threshold for peptides imported from the MaxQuant msms.txt output table was set to 0.01, and all other settings were left to default. Analysis of DIA data from the experimental samples was done in Spectronaut using our DDA spectral library and the default settings. Across-run normalization based on total peak areas was performed by Spectronaut. Peptide abundances were exported as a Spectronaut report and further processed using the R language for statistical computation, version: 3.4.4 [56]. For a total overview of the studied samples, peptides were selected using a quality value cut-off condition of ≤ 10^–3^ in 50% of samples (this filter was applied per sample fraction). For further pairwise statistical comparison between conditions, peptides in each sample fraction were selected using a quality value cut-off condition of ≤ 10^–3^ in 50% of the samples of one condition. Peptide abundances were computed by summation of the peak area of the top two abundant fractions, preceded by peptide normalization using the *normalizeCyclicLoess* function from the limma R package, which was set to ‘fast’ and iterations were set to 3. Protein abundances were computed by summation of the normalized abundancies of the top five most abundant peptides for a respective protein.

### Statistical analysis of differential protein expression

Differential expression analysis between conditions was performed on log-transformed protein abundances. Permutation-based modified t-statistics with multiple testing correction by False Discovery Rate (FDR) was applied using the *SAM* function from the siggenes R package, using the method ‘d.stat’, running 1000 permutations. An FDR adjusted *q*-value threshold of 0.05 was used to discriminate proteins of interest after differential expression analysis.

### Cell type enrichment analysis

Cell type enrichment analysis can help to stratify data from mixed cell populations, without the need for physical cell sorting [4, 45]. This is frequently applied on bulk RNA expression data, but to our knowledge has not been used for protein expression data yet. As single cell proteomic data sets are for now unattainable, we set out to identify cell type enrichment in our FTD protein signatures based on published transcriptomic profiles -assuming that proteins are detected in cells that express the corresponding genes. Cell type enrichment analysis of differentially expressed proteins was based on several relevant and extensive human brain single cell transcriptome data resources. For analysis of our frontal cortical protein signatures, single nuclei RNAseq data of 10,319 cells from post-mortem frontal cortical tissue of four adult controls was used [39]. For analysis of temporal cortical protein signatures, a combination of single cell RNAseq data of 466 cells from eight adult control donors [14] and single nuclei RNAseq data of 15,928 cells from eight adult control donors [30] from temporal cortical tissue of either surgical procedures or post-mortem, was used.

Pre-processing and analysis of single cell and single nuclei RNAseq data sets was performed using the Python package Scanpy (version 1.5.1) [74] as described previously [46]. In short, cell-gene matrices were filtered for outliers and gene expression was normalized per cell. All cells were clustered using Louvain clustering implementation [69] on the top 1000 highly variable genes. To identify cell types, marker genes and expected cell types were inferred from the original publications of the data sets. Briefly, angiotensinogen (*AGT*), electrogenic sodium bicarbonate cotransporter 1 (*SLC4A4*), and excitatory amino acid transporter 2 (*SLC1A2*) were taken as markers for astrocytes, vascular endothelial growth factor receptor 1 (*FLT1*), dual specificity protein phosphatase 1 (*DUSP1*), and nostrin (*NOSTRIN*) for endothelial cells, vesicular glutamate transporter 1 (*SLC17A7*) for excitatory neurons, glutamate decarboxylase 1 (*GAD1*) for inhibitory neurons, amyloid beta A4 precursor protein-binding family B member 1-interacting protein (*APBB1IP*) and TYRO protein tyrosine kinase-binding protein (*TYROBP*) for microglia, myelin-associated oligodendrocyte basic protein (*MOBP*) for oligodendrocytes, and protocadherin-15 (*PCDH15*) and platelet-derived growth factor receptor alpha (*PDGFRA*) as markers for oligodendrocyte precursor cells (OPCs). Clusters that could not be clearly identified with one cell type were labelled ‘unknown’.

Normalized gene expression data and cell type label matrices were subsequently used for expression-weighted cell type enrichment analysis using the EWCE package, version 1.2.0 [65] in R. The total set of DIA quantified proteins for both FTD subtypes was used as the background set, from which 20,000 random lists were generated for bootstrapped analysis of the probability distribution of cell type expression. In addition, proteins that showed specificity values ≥ 0.5 for a certain cell type were considered as highly enriched for that cell type. Furthermore, to look into synapse enriched proteins, we used the knowledgebase SynGO (version: 20180731) [35]. Proteins that were annotated within SynGO were considered enriched for the synapse, with a division into pre- and postsynaptic based on ontology structure.

### Overrepresentation analysis

Gene ontology (GO) enrichment was performed using g:Profiler web server (version: rev 1760 e93 eg40) [58], with all settings on default, g:Profiler-based multiple testing correction (g:SCS method), and with the total of DIA quantified proteins as background for both FTD subtypes. Classical GO terms, i.e. biological process (BP), cellular component (CC), and molecular function (MF) were examined, taking only terms containing five or more proteins into account. When possible, GO terms were further organized into GO groups in keeping with shared functions and proteins. For visualization, only ‘Best Per Parent’ GO terms are shown. These were selected by hierarchical filtering (moderate), where for every parent GO term its sibling term with the strongest p-value was chosen. For detailed analysis regarding affected synaptic processes we used SynGO (version: 20180731) [35], with FDR-based multiple testing correction and the total of DIA quantified proteins as background for both FTD subtypes. For detailed analysis on affected mitochondrial processes we used the human MitoCarta inventory (version 3.0) [57] in combination with the PANTHER Classification System (version 14.0) [47]. Differentially expressed proteins that were annotated to the mitochondrion according to MitoCarta were analysed for GO enrichment using the PANTHER Overrepresentation Test, with a Fisher's Exact test and FDR-based multiple testing correction, and with the total set of MitoCarta annotated proteins detected within the DIA quantified proteins as background.

### Immunoblotting for validation of differential protein expression

Post-mortem middle frontal gyrus and middle temporal gyrus cortical tissues from a random subset of patients and controls (n = 8/group) were selected from the original study cohort. Additionally, frontal and temporal cortex tissues from newly confirmed genetic FTD-GRN (n = 2) and FTD-MAPT (n = 3) cases were requested from the Netherlands Brain Bank, Netherlands Institute for Neuroscience, Amsterdam, to use as an independent validation cohort (Table 2).

**Table 2:** Demographic, clinical, and post-mortem characteristics for cortical frontal and temporal samples from the independent validation cohort

	FTD-GRN (N = 2)		FTD-MAPT (N = 3)	
*Demographics*				
Female, n (%)	2/2 (100.0)		0/3 (0.0)	
Age, median (range)	63.5 (61–66)		57.0 (55–65)	
*Clinical characteristics*				
Genetic mutation (n)	S82fs (1)		A886G (1)	
	c.1179 + 104_1536delinsCTGA (1)		P301L (2)	
Disease pathology (n)				
• Atypical Pick’s disease	–		1	
• Tau	–		2	
• TDP-type A	2		–	
*Post-Mortem* *determinants*				
Post-mortem delay, median (range)	08:05 (05:19–10:50)		05:15 (04:25–05:40)	
Cerebrospinal fluid pH, median (range)	6.23 (6.22–6.24)		6.36 (6.35–6.37)	
Brain weight in grams, median (range)	1027 (1002–1052)		1197 (1100–1395)	
Braak score, median (range)	1 (n/a)		0 (0–0)	

ApoE profile information was unknown for these cases. n/a; not available

Protein extracts for immunoblotting were prepared by lysis of whole cortical tissue in Laemmli reducing SDS sample buffer using a 1:20 tissue weight to lysis buffer ratio. Proteins were denatured at 98 °C for 5 min, with the exception of samples used for immunoblotting of mitochondrial proteins, which were denatured at 50 °C for 5 min. Proteins were separated by SDS-PAGE using Criterion™ TGX stain-free™ precast gels (Bio-Rad, Hercules, CA, USA) and transferred (40 V o/n at 4 °C) onto a 0.45 µm PVDF membrane (Merck Millipore), which was pre-incubated in 100% methanol. Membranes were blocked with 5% non-fat milk (Sigma-Aldrich, St. Louis, MO, USA), incubated with primary antibody at RT for 2 h and then with matching HRP-conjugated secondary antibodies at RT for 1 h (Agilent Dako, Santa Clara, CA, USA). After washing, the membranes were scanned with Femto ECL Substrate (Thermo Fisher Scientific, Waltham, MA, USA) using the Odyssey Fc system (LI-COR Bioscience, Lincoln, NA, USA). Images were quantified using Image Studio Lite software (version 2.0.38). Differences in loading were corrected using the quantification of the total protein load, which was visualized using a chemidoc EZ (Bio-Rad), and immunoblot signals were normalized to NDC samples. The following primary antibodies were used: total OXPHOS rodent WB antibody cocktail (1/1000, Abcam, ab110413) and anti-myelin proteolipid protein (1/1000, Serotec, MCA839G).

### Alzheimer’s disease proteomics

The cortical temporal FTD-MAPT protein profile was compared with a cortical temporal Alzheimer’s disease (AD) protein profile. Proteomic data was taken from a subset of samples originating from the post-mortem brain cohort of the *100-plus Study*, a research initiative focussing on the mechanisms of healthy aging. From this cohort, containing NDCs, AD patients, and healthy centenarians, we selected post-mortem middle temporal gyrus tissue from 10 sporadic AD cases with Braak tau score ≥ 5 and 10 non-demented controls. All tissues came from the Netherlands Brain Bank, Netherlands Institute for Neuroscience, Amsterdam, and were age and sex matched with the temporal FTD-MAPT samples (Table 3).

**Table 3:** Demographic, clinical, and post-mortem characteristics for cortical temporal samples from the sporadic AD cohort

	NDC (N = 10)	AD (N = 10)
*Demographics*		
Female, n (%)	6/10 (60.0)	5/10 (50.0)
Age, median (range)	70.0 (57–75)	64.5 (62–67)
*Clinical characteristics*		
ApoE profile (n)		
• 32	2	2
• 33	3	4
• 42	1	–
• 43	–	4
• 44	–	–
• Unknown	4	–
*Post-mortem determinants*		
Post-mortem delay, median (range)	07:23 (05:30–09:35)	05:55 (04:10–07:30)
Cerebrospinal fluid pH, median (range)	6.51 (6.03–7.20)	6.42 (6.35–6.75)
Brain weight in grams, median (range)	1188 (1153–1339)	1011 (790–1254)
Braak score, median (range)	1 (0–2)	6 (5–6)

Tissue was processed as described above, with a few alterations; an equal volume of 0.5 × 10^9^ µm^3^ of grey matter tissue was collected for each sample. Proteins were size separated on 10% Bis–Tris acrylamide gels using 1.5 M Tris/Glycine SDS running buffer pH 8.3. Gels were fixed overnight and shortly stained with colloidal Coomassie Blue G-250 the next morning. After destaining, trypsin digestion, and peptide extraction, samples were dried and dissolved again with 100 µl Mobile phase A (2% acetonitrile/0.1% formic acid) to be cleaned using the OASIS filter plate according to protocol (Waters Chromatography Europe BV, Etten-Leur, The Netherlands). DIA proteomics data was obtained using an identical approach, with the exception of gel fractionation. A spectral library was prepared using peptides from pooled protein extracts from a mix of NDC, AD, and centenarian samples, which identified 4,948 protein groups by MaxQuant search (version 1.6.3.4) against the human proteome using the UniProt FASTA (release May 2018) and Biognosys iRT FASTA databases. Data extraction and analysis, and statistical comparison of NDC *vs* AD cases was performed using the same methods.

## Results

### Cohort description

Cortical tissues from the middle frontal gyrus and middle temporal gyrus were collected from nine FTD-GRN brains, 13 FTD-MAPT brains, and 11 NDC brains. Both patient groups had significantly lower ages than NDCs and their post-mortem brain weights and CSF pH values were lower (Table 1). Neuropathological examination (see images in Additional File 1) revealed that all FTD-GRN cases exhibited TDP-43 immunoreactivity in both cortical areas, consistent with TDP-subtype A. All FTD-MAPT cases were characterized by tau-positive neuronal inclusions, neuropil threads, and tangles in both cortical areas, with *MAPT*-variant specific features.

### Proteomic analysis of FTD-GRN and FTD-MAPT shows brain region-specific protein expression

Using DIA LC–MS/MS we measured abundances of 22,995 unique peptides. Applying our quality value cut-off on all samples together, yielded 9,545 unique peptides, mapping to 2,040 unique proteins measured. Analysis of technical replicates showed a median coefficient of variation of 0.13 in protein abundances, indicating high reproducibility between samples (Additional File 2). For further statistical analysis, quality value cut-off selection of peptides was performed for single disease comparisons (FTD *vs* NDC) and frontal and temporal samples separately (Table 4).

**Table 4:** Number of unique peptides and proteins measured within the FTD cohort

Brain area	Group	QC Condition	Peptides (n)	Proteins (n)
Frontal, Temporal	NDC, FTD-GRN, FTD-MAPT	50% of all samples	9,545	2,040
Frontal	NDC, FTD-GRN	50% of NDC or FTD-GRN	12,939	2,400
	NDC, FTD-MAPT	50% of NDC or FTD-MAPT	11,958	2,346
Temporal	NDC, FTD-GRN	50% of NDC or FTD-GRN	9,425	2,037
	NDC, FTD-MAPT	50% of NDC or FTD-MAPT	9,123	2,010

Peptides and proteins are selected using quality value filtering on peptide level (see methods for details). Quality value peptide selection separated on brain area and FTD subtype gave the opportunity to analyse the highest number of proteins per statistical comparison

**Fig. 2:**
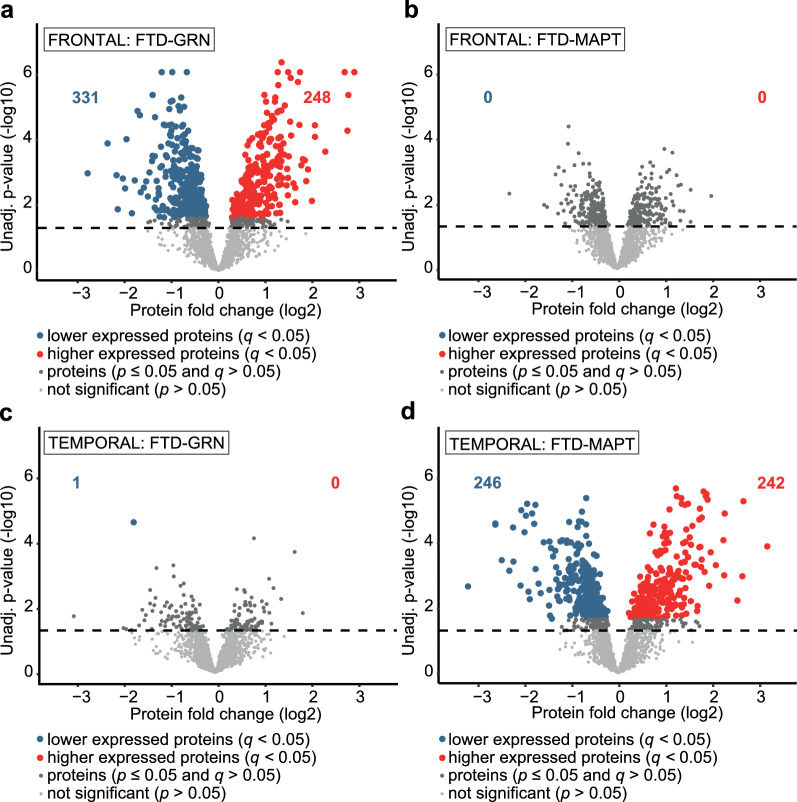
Differential protein expression in most-affected areas for genetic FTD subtypes shows brain region-specific protein signatures. **A** Differential protein expression (at *q* < 0.05) in frontal cortical tissue for FTD-GRN *vs* NDC. **B** Differential protein expression (at *q* < 0.05) in frontal cortical tissue for FTD-MAPT *vs* NDC. **C** Differential protein expression (at *q* < 0.05) in temporal cortical tissue for FTD-GRN *vs* NDC. **D** Differential protein expression (at *q* < 0.05) in temporal cortical tissue for FTD-MAPT *vs* NDC. Differential expression analysis was done using permutation-based modified t-statistics. An FDR-adjusted *q*-value threshold of 0.05 was used to discriminate proteins of interest. The number of differentially expressed proteins is extensive in both FTD-GRN (n = 580) and FTD-MAPT (n = 488) cases

Significant differential protein expression (Fig. 2, Table 5, and Additional File 3) for FTD-GRN *vs* NDC was mainly found for frontal cortex (579 proteins), and almost absent in temporal cortex (one protein) (*q* < 0.05). In contrast, for FTD-MAPT *vs* NDC, significant differential protein expression was found in temporal cortex (488 proteins), and not in frontal cortex (*q* < 0.05). The top 50 significantly differentially expressed proteins with the largest fold change are listed for both subtypes in Table 6. The differential expression of several well-known neurodegeneration-related proteins (e.g. glial fibrillary acidic protein (GFAP) and MAPT) is highlighted for the most-affected brain region of both subtypes in Additional File 3.

**Table 5:** Number of significantly differentially expressed proteins within the FTD cohort

Brain area	Disease comparison (n:n)	*p* < 0.05 (n)	*q* < 0.05 (n)
Frontal	NDC *vs* FTD-GRN (11:8)	762	579
	NDC *vs* FTD-MAPT (11:11)	388	0
Temporal	NDC *vs* FTD-GRN (8:9)	170	1
	NDC *vs* FTD-MAPT (8:13)	655	488

Results of differential expression analysis are shown both at non-corrected (*p* < 0.05) and multiple comparison corrected (*q* < 0.05) statistical cut-offs

**Table 6:** The top 50 significantly differentially expressed proteins in FTD-GRN *vs* NDC and FTD-MAPT *vs* NDC

	Frontal cortical tissue of FTD-GRN vs NDC			Temporal cortical tissue of FTD-MAPT vs NDC		
Direction of differential expression	Gene Symbol	Fold Change	Statistical *q* value	Gene Symbol	Fold Change	Statistical *q* value
Higher	PHYHD1	7.434	0.0001099	SUCLG2	9.226	0.0021797
Higher	ICAM1	6.803	0.0003298	GFAP	6.468	0.0005181
Higher	CD44	6.718	0.0011453	H1-0	6.366	0.0083540
Higher	GFAP	6.439	0.0001099	CD44	5.912	0.0221017
Higher	TNC	4.843	0.0025774	FAT3	4.893	0.0006696
Higher	PPIA	4.158	0.0013982	PHYHD1	4.857	0.0079645
Higher	FAT3	4.156	0.0008582	TNC	4.800	0.0019158
Higher	GSTM5	3.974	0.0233293	ITGA7	4.289	0.0053771
Higher	BLVRB	3.748	0.0055936	GSTM5	3.971	0.0028718
Higher	SORD	3.653	0.0092284	SDC3	3.882	0.0123535
Higher	GJA1	3.579	0.0037439	PLSCR4	3.795	0.0005181
Higher	ARHGDIB	3.473	0.0035394	TNS3	3.731	0.0005181
Higher	HIKESHI	3.399	0.0048696	GSTM3	3.692	0.0005181
Higher	CSRP1	3.336	0.0001099	PLCD3	3.624	0.0069908
Higher	FKBP1A	3.304	0.0008582	PYGM	3.569	0.0005181
Higher	PLSCR4	3.232	0.0001799	PPIA	3.479	0.0008116
Higher	ARHGDIA	3.107	0.0127071	PRODH	3.472	0.0041525
Higher	ISYNA1	3.104	0.0248770	VAMP5	3.443	0.0058829
Higher	AKR1B1	3.024	0.0101091	CSRP1	3.407	0.0022148
Higher	CBR1	2.924	0.0013718	HEPACAM	3.378	0.0006236
Higher	GSTM2	2.904	0.0001484	EGFR	3.353	0.0022489
Higher	PNP	2.882	0.0008582	GJA1	3.352	0.0008976
Higher	TPR	2.821	0.0031321	NQO1	3.267	0.0275547
Higher	HEPACAM	2.798	0.0001099	FKBP1A	3.244	0.0338166
Higher	GSPT1	2.791	0.0080283	SNTB1	3.180	0.0053499
Lower	CEP128	0.145	0.0066177	HNMT	0.107	0.0125724
Lower	LPCAT4	0.194	0.0017604	ERMN	0.160	0.0009540
Lower	RDH13	0.222	0.0072139	MOG	0.161	0.0009540
Lower	CPLX1	0.226	0.0346641	OPALIN	0.176	0.0042780
Lower	UBAP2L	0.244	0.0082093	PANK4	0.198	0.0066545
Lower	CACNG8	0.252	0.0129590	MOBP	0.209	0.0010363
Lower	FBXL18	0.257	0.0014650	JAM3	0.210	0.0046333
Lower	CACNG3	0.278	0.0411538	PLLP	0.234	0.0122430
Lower	PEX16	0.291	0.0089505	STAT1	0.236	0.0006236
Lower	HABP4	0.303	0.0005289	MBP	0.248	0.0013509
Lower	SNCG	0.313	0.0006494	MAG	0.253	0.0007440
Lower	MRPS36	0.317	0.0157407	MAP6D1	0.258	0.0005181
Lower	TIMM13	0.332	0.0126697	DBNL	0.263	0.0152205
Lower	NIPSNAP3B	0.344	0.0093255	HSPA12B	0.268	0.0212848
Lower	MAG	0.352	0.0188804	SNCG	0.278	0.0006696
Lower	UBQLN1	0.361	0.0063254	CPLX1	0.286	0.0009540
Lower	IGSF9B	0.375	0.0038853	LGI3	0.291	0.0005181
Lower	KIAA1211L	0.378	0.0031165	HABP4	0.292	0.0064298
Lower	SYNPO	0.378	0.0003298	CPNE5	0.305	0.0115622
Lower	NDUFV3	0.390	0.0324149	CCAR1	0.315	0.0165266
Lower	TFRC	0.390	0.0031321	SIRT2	0.330	0.0019158
Lower	TAX1BP1	0.392	0.0006785	TIMM9	0.357	0.0271267
Lower	KATNAL1	0.397	0.0219467	RTN4RL2	0.359	0.0143297
Lower	VAMP4	0.401	0.0091035	PLP1	0.360	0.0019158
Lower	MAP7D1	0.402	0.0361734	SULT4A1	0.363	0.0425741

The top 25 higher and top 25 lower significant differentially expressed proteins (*q* < 0.05) with the largest fold changes are listed for frontal cortical samples of FTD-GRN *vs* NDC and for temporal cortical samples of FTD-MAPT *vs* NDC

### Cell type enrichment analysis reveals cell-specific involvement in FTD-GRN and FTD-MAPT

**Fig. 3:**
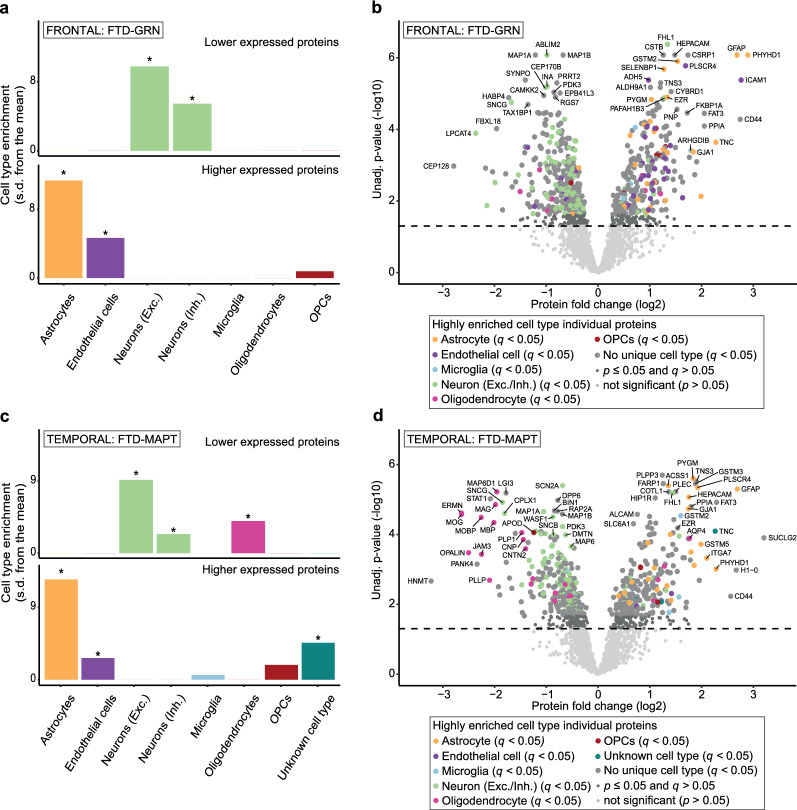
EWCE analysis on differentially expressed proteins shows cell type specific involvement in genetic FTD subtypes. **A** EWCE analysis of lower (n = 331) and higher (n = 248) expressed proteins in frontal cortical FTD-GRN *vs* NDC. **C** EWCE analysis of lower (n = 246) and higher (n = 242) expressed proteins in temporal cortical FTD-MAPT *vs* NDC. EWCE analysis is performed using bootstrapped t-test statistics with a multiple testing correction done by the Benjamini–Hochberg method. Comparison of EWCE analysis results between FTD-GRN and FTD-MAPT shows astrocyte (both *p* = 0) and endothelial cell (*p* = 0 and *p* = 0.0133, respectively) enrichment for higher expressed proteins, and excitatory (both *p* = 0) and inhibitory (*p* = 0 and *p* = 0.0264) neuronal cell type enrichment for lower expressed proteins in both FTD subtypes. In FTD-MAPT, distinct oligodendrocyte enrichment (*p* = 0.0004) is observed for lower expressed proteins. **(B,D)** Proteins that are highly enriched for specific cell types (see methods) show the extent of differential protein expression ongoing in these cell types. In these plots, cell type specificity values for excitatory and inhibitory neurons are summed per protein. Exc.; excitatory, Inh.; Inhibitory, s.d.; standard deviation

EWCE analysis of significantly differentially expressed proteins demonstrated distinct cell type involvement for FTD-GRN and FTD-MAPT (Fig. 3). In both, higher expressed proteins showed enrichment for astrocytes and endothelial cells, and lower expressed proteins showed enrichment for neurons. In FTD-MAPT, lower expressed proteins showed additional enrichment for oligodendrocytes.

As these cell type-specific protein expression patterns might be due to changes in cell numbers, protein abundances associated with a particular cell type could also indicate cell loss or gain instead of specific protein regulation. Therefore, we analysed the fold changes between FTD and NDC of all highly-enriched cell type-specific proteins. This showed that the majority of these proteins is normally distributed within the range of NDC protein variation, with values ranging from negative (lower expressed) to positive (higher expressed), and that significantly differentially expressed proteins form a separate population within this distribution (see analysis in Additional File 4). This demonstrates that cell type-specific changes in protein expression are unlikely to be merely the result of cell ratio changes. Taken together, EWCE analysis shows that differential protein expression in genetic FTD subtypes is partly linked to distinct cell types.

### GO analysis identifies distinct biological processes involved in FTD-GRN and FTD-MAPT

**Fig. 4:**
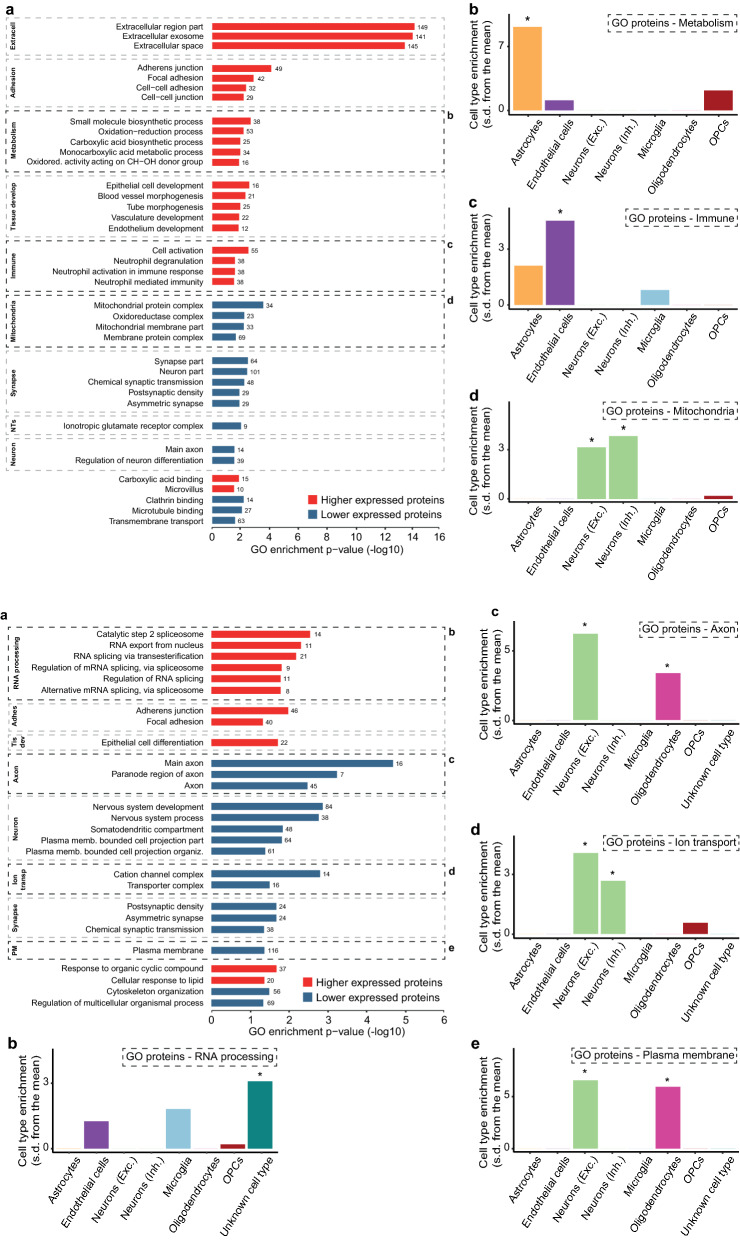
Figure 4-I GO analysis shows distinct involvement of metabolic and immune processes and mitochondrial functioning in FTD-GRN. **A** GO term enrichment analysis on lower (n = 331) and higher (n = 248) differentially expressed proteins in frontal cortical FTD-GRN *vs* NDC. Only classical GO terms (BP/CC/MF) are taken into account, and only ‘Best Per Parent’ GO terms are shown. The number of proteins in each term is listed. GO terms are further categorized into GO groups. ‘Metabolism’, ‘Immune’, and ‘Mitochondria’ GO groups are distinctly present in FTD-GRN when compared with FTD-MAPT. **B** EWCE analysis of the ‘Metabolism’ GO group shows the involvement of astrocytes (*p* = 0) in these processes. **C** EWCE analysis of the ‘Immune’ GO group shows the involvement of endothelial cells (*p* = 0.00035) in these processes. **D** EWCE analysis of the ‘Mitochondria’ GO group shows enrichment for both excitatory and inhibitory neurons (*p* = 0.0056 and *p* = 0.0049, respectively). Figure 4-II GO analysis shows distinct involvement of RNA processing, axons, ion transport, and the plasma membrane in FTD-MAPT. **A** GO term enrichment analysis on lower (n = 246) and higher (n = 242) differentially expressed proteins in temporal cortical FTD-MAPT *vs* NDC. Only classical GO terms (BP/CC/MF) are taken into account, and only ‘Best Per Parent’ GO terms are shown. The number of proteins in each term is listed. GO terms are further categorized into GO groups. ‘RNA processing’, ‘Axon’, ‘Ion transport’, and ‘Plasma membrane’ GO groups are distinctly present in FTD-MAPT when compared with FTD-GRN. **B** EWCE analysis of the ‘RNA processing’ GO group shows no specific cell type enrichment. **C** EWCE analysis of the ‘Axon’ GO group shows involvement of excitatory neurons (*p* = 0) and oligodendrocytes (*p* = 0.0056). **D** EWCE analysis of the ‘Ion transport’ GO group shows enrichment for both excitatory and inhibitory neurons (*p* = 0.001 and *p* = 0, respectively). **E** EWCE analysis of the ‘Plasma membrane’ GO group shows involvement of excitatory neurons (*p* = 0) and oligodendrocytes (*p* = 0). EWCE analysis is performed using bootstrapped t-test statistics with a multiple testing correction done by the Benjamini-Hochberg method. Adhes.; adhesion, Develop.; development, Extracell.; extracellular, Exc.; excitatory, Inh.; Inhibitory, Memb.; membrane; NTs; Neurotransmitters, Oxidored.; oxidoreductase, PM; plasma membrane, Tis dev.; tissue development, Transp.; transport, s.d.; standard deviation

To determine which biological processes are affected in FTD, we used GO analysis on the differentially higher and lower expressed proteins separately (see extensive results in Additional File 5). ‘Best Per Parent’ GO terms, further categorized into GO groups, are shown for FTD-GRN in Fig. 4-IA and for FTD-MAPT in Fig. 4-IIA.

FTD-GRN and FTD-MAPT showed overlap in GO groups reflecting processes that may be generally affected in FTD. Higher expressed proteins overlapped for ‘Tissue development’, ‘Cell adhesion’, and ‘Extracellular space’, and lower expressed proteins overlapped for ‘Neuron’ and ‘Synapse’, pointing towards a shared impairment of neuronal function and connectivity. In-depth SynGO analysis shows that differentially expressed synaptic proteins in FTD-GRN and FTD-MAPT are localized in presynaptic and postsynaptic compartments, and are implicated in a wide range of functions (see visualization in Additional File 6 and extensive results in Additional File 7), likely reflecting the overall impact of neurodegeneration on the synapse.

In FTD-GRN only, higher expressed proteins are enriched for GO terms related to metabolism and the immune system (Fig. 4-IA). EWCE analysis of ‘Metabolism’ proteins shows enrichment for astrocytes (Fig. 4-IB), and ‘Immune’ proteins are enriched for endothelial cells (Fig. 4-IC). Furthermore, distinct for FTD-GRN, prominent enrichment of mitochondria-related GO terms for lower expressed proteins is observed. A total of 85 proteins point towards affected mitochondria. Detailed dissection of the mitochondrial regulation shows specific enrichment for the oxidoreductase complex (see Additional File 8), suggesting specific functional alterations rather than overall (structural) downregulation or loss of mitochondria in FTD-GRN. Especially respiratory chain complex I (RCCI) seems to be affected, with 12 of in total 38 RCCI proteins measured in our data set (32%) lower expressed in FTD-GRN, and only four out of 35 RCCI proteins measured in FTD-MAPT differentially expressed. EWCE analysis of ‘Mitochondria’ proteins shows enrichment for neurons (Fig. 4-ID), indicating that these cells might be particularly affected in FTD-GRN.

In FTD-MAPT only, higher expressed proteins are enriched for GO terms related to RNA processing. EWCE analysis of ‘RNA processing’ proteins showed no specific involvement of cell types (Fig. 4-IIB). Lower expressed proteins in FTD-MAPT showed distinct enrichment for ‘Axon’, ‘Ion transport’, and ‘Plasma membrane’. EWCE analysis on ‘Axon’ (Fig. 4-IIC) and ‘Plasma membrane’ proteins (Fig. 4-IIE) alluded to the involvement of oligodendrocytes. A comparison of statistical effect sizes of all GO group proteins distinct for FTD-GRN and FTD-MAPT confirms that these biological processes are indeed strongly biased towards their respective FTD subtype (see visualizations in Additional File 9 and Additional File 10).

In the process of selecting targets for validation study, proteins were chosen for their biological relevance and distinct regulation in only one FTD subtype. Immunoblotting of selected target proteins for FTD-GRN and FTD-MAPT was carried out on a random subset of samples from our cohort as well as on samples from an independent cohort (Table 2). Results confirm lower expression of mitochondrial RCC proteins for subunits I-IV in FTD-GRN only, though detected differences are not statistically significant per protein. PLP1 expression, which was lower in FTD-MAPT specifically according to our mass spectrometry data, indeed shows a strong trend (*p* = 0.0595) for decreased expression in FTD-MAPT (see Additional File 11 for all immunoblotting results). Raw images of gels and immunoblots can be found in Additional File 12.

Taken together, GO analysis illustrates both the presence of general neurodegenerative processes and subtype-distinct biological processes for FTD-GRN and FTD-MAPT, with a cell type-specific involvement in many of these processes.

### Comparing FTD-MAPT with AD confirms both distinct and general neurodegenerative protein signatures

**Fig. 5:**
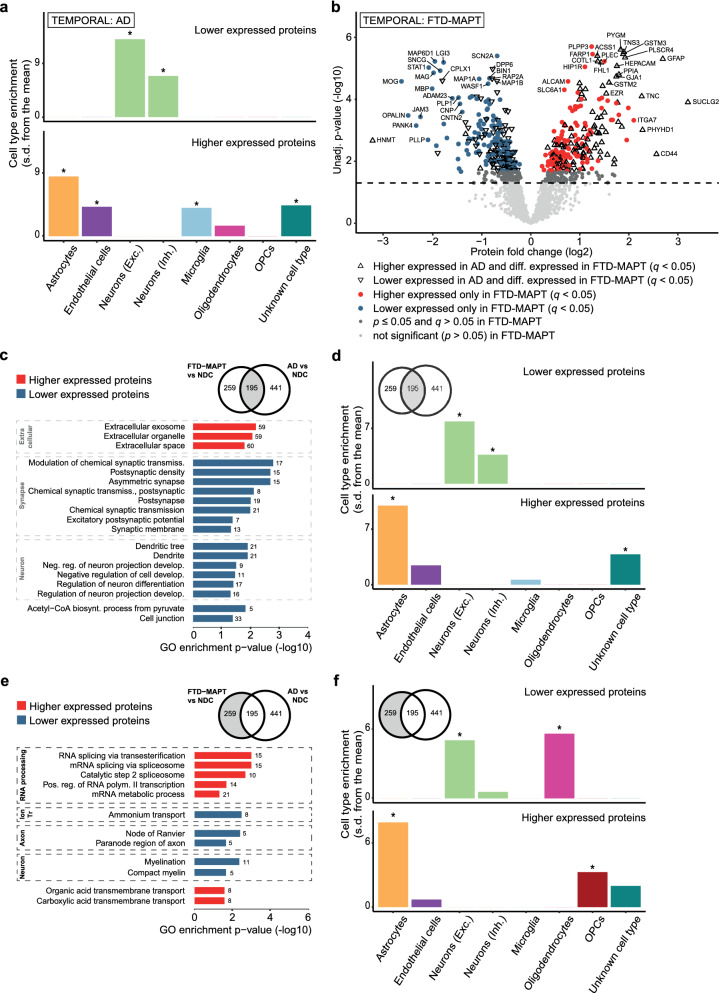
Comparison of profiles between FTD-MAPT and AD demonstrates presence of FTD subtype-specific and general neurodegenerative protein signatures. **A** EWCE analysis of lower (n = 406) and higher (n = 556) expressed proteins in temporal cortical AD *vs* NDC shows astrocyte, endothelial cell, and microglial cell enrichment (all *p* = 0) for higher expressed proteins, and excitatory and inhibitory neuron enrichment (both *p* = 0) for lower expressed proteins. Comparison with EWCE results for FTD-MAPT confirms distinct involvement of oligodendrocytes in FTD-MAPT. **B** Overlay of differentially expressed proteins shared between FTD-MAPT and AD (n = 195) on the protein profile for FTD-MAPT shows that the majority of shared proteins have the same direction of differential expression. **C** GO analysis on shared proteins. **D** EWCE analysis on shared proteins. Significant enrichment is seen for astrocytes (*p* = 0) for higher expressed proteins, and for excitatory (*p* = 0) and inhibitory (*p* = 0.002) neurons for lower expressed proteins. **E** GO analysis on proteins that are only differentially expressed in FTD-MAPT (n = 259). ‘RNA processing’, ‘Ion transport’, ‘Axon’ and ‘Neuron’ GO groups are confirmed to be distinct for the FTD-MAPT subtype when compared with AD. **F** EWCE analysis on proteins only differentially expressed in FTD-MAPT. Significant enrichment is seen for astrocytes (*p* = 0) and OPCs (*p* = 0.0074) for higher expressed proteins, and for excitatory neurons and oligodendrocytes (both *p* = 0) for lower expressed proteins. EWCE analysis is performed using bootstrapped t-test statistics with multiple testing correction by the Benjamini–Hochberg method. For GO analysis, only classical GO terms (BP/CC/MF) are taken into account and only ‘Best Per Parent’ GO terms are shown. The number of proteins in each GO term is listed. GO terms are further categorized into GO groups. Biosynth.; biosynthesis, Exc.; excitatory, Develop.; development, Inh.; Inhibitory, Ion tr; ion transport, Neg. reg.; negative regulation, Polym.; polymerase, s.d.; standard deviation, Transmiss.; transmission

Tau pathology is a shared disease hallmark between FTD-MAPT and AD. Comparison of differential protein expression in the same brain area between the two might help identify proteins implicated in shared neurodegenerative mechanisms, as well as proteins representing distinct aspects of pathological mechanisms for both diseases. For this, we used a temporal cortical AD *vs* NDC proteomic data set in which we quantified 3,332 unique proteins, of which 962 were significantly differentially expressed compared to NDC (*q* < 0.05) (see Additional File 13). When comparing the 1,847 proteins that were quantified in both the FTD-MAPT and AD datasets (also Additional File 13), 195 differentially expressed proteins were shared, and 259 differentially expressed proteins were distinct for FTD-MAPT. The majority of shared proteins have the same direction of differential expression (Fig. 5B).

When comparing EWCE results between AD (Fig. 5A) and FTD-MAPT (Fig. 3C), a shared association of astrocytes with higher expressed proteins, and of neurons with lower expressed proteins was revealed, indicating more common neurodegenerative processes leading to astrogliosis and neurodegeneration as shared mechanisms. Significant endothelial and microglial enrichment is seen in AD, whereas oligodendrocyte involvement is specific for FTD-MAPT, suggesting that these cell types specifically contribute to AD and FTD-MAPT pathology, respectively.

GO analysis of the 195 proteins shared between FTD-MAPT and AD (Fig. 5C and Additional File 14) highlighted GO groups ‘Neuron’ and ‘Synapse’ linked to lower expressed proteins. SynGO analysis showed that the 55 shared differentially expressed synaptic proteins are implicated in a wide range of functions (extensive results seen in Additional File 15). In addition, EWCE analysis demonstrated that shared processes are indeed enriched for astrocytes and neurons (Fig. 5D).

GO analysis of the 259 distinct FTD-MAPT proteins revealed distinct enrichment of ‘RNA processing’ GO terms for higher expressed proteins, and ‘Axon’ GO terms, specifically indicating the myelin sheath, for lower expressed proteins (Fig. 5E). EWCE analysis confirmed the subtype-specific involvement of oligodendrocytes in FTD-MAPT and additionally identified oligodendrocyte precursor cells (OPC) as a cell type involved in FTD-MAPT pathology (Fig. 5F). OPCs were not significantly enriched previously (Fig. 3C), demonstrating the power of excluding general neurodegeneration-associated proteins through filtering with the AD protein set.

Thus, comparison of FTD-MAPT with AD aided in delineating common neurodegenerative mechanisms as well as FTD subtype-specific changes, including the specific involvement of certain brain cell types.

## Discussion

This study describes the differential proteins expression in cortical frontal and temporal regions of the brain in FTD-GRN and FTD-MAPT. Region-specific protein signatures indicated the involvement of cell type-specific distinct biological processes in these FTD subtypes. Importantly, comparing FTD-MAPT to AD revealed overlapping neurodegenerative processes as well as the existence of FTD-MAPT-specific disease mechanisms.

The occurrence and extent of differential protein expression for both subtypes reflect known brain atrophy patterns. In FTD-GRN, atrophy is usually found in a diffuse hemispheric manner, including in frontal and temporal lobes [10, 62]. Six FTD-GRN patients, for which we had neuropathological reports, presented with severe frontal and little to no temporal atrophy, explaining the frontal focus of their differential protein expression. FTD-MAPT patients are characterized by predominant temporal lobe atrophy [10, 62], which is reflected by the extensive differential protein expression specifically in the temporal cortex of our cases.

A clear indication that disease-specific involvement of cell types and biological processes in neurodegeneration exists, even in end-stages, came from comparison with a protein signature of AD. Although both FTD subtypes and AD share involvement of astrocytes and neurons, there was clear discrepancy in microglial enrichment. Although microglia have been implicated in FTD [24], significant microglial enrichment was absent in this study. A recent investigation in FTD demonstrated variable involvement of microglia according to brain region and subtype, with a higher burden in white versus grey matter [75]. As our study specifically analysed grey matter tissue, this could explain the lack of microglial enrichment. Further comparison between FTD-MAPT and AD confirmed the existence of both shared general neurodegenerative as well as FTD-MAPT-specific processes.

In FTD-GRN, we found distinct involvement of immune-related processes, primarily linked to endothelial cells. Involvement of endothelial cells in FTD has only been described incidentally, mainly in relation to blood–brain-barrier (BBB) pathology [32, 33, 52]. Their possible active involvement in immune processes is a new finding. The increased expression of intercellular adhesion molecule 1 (ICAM1; 6,80x) hints towards a role for the traversing of leukocytes across the BBB, which appears to be affected more by ICAM1 expression than inflammatory molecules or BBB integrity [1, 41]. This finding is strengthened by multiple GO terms pointing towards the presence of activated peripheral immune cells and an ongoing immune response. Discernment of the specific response type is difficult, as higher expression of proinflammatory (e.g. PPIA), anti-inflammatory (e.g. ANXA1 and ASAH1), and ambiguous inflammatory proteins (e.g. CD44 and PRDX1/6) was detected. Further research is needed to characterize the precise role of the immune response in FTD-GRN. Neuroinflammation is increasingly implicated in FTD disease progression [7, 8]. A recent proteomic study of frontal cortex tissue from FTD-TDP patients identified an ‘inflammatory’ protein module enriched for astrocytes and microglia [70]. The fact that we identify endothelial cells at the forefront of immune-related processes in our FTD-GRN cohort might be specifically related to the genetic cause of FTD in these cases. This is illustrative of the strength of our approach to specifically select genetic cases, which limits possible interference of biological processes related to sporadic FTD.

We also identified a striking pattern of mitochondrial dysregulation, suggesting specific functional alterations of mitochondria in FTD-GRN. This finding is consistent with Umoh et al*.*, in which decreased expression of a mitochondrial module in frontal cortex tissue from FTD-TDP patients was reported [70]. Although mitochondrial dysfunction seems to be a common denominator of neurodegeneration [20, 49], the differential expression of RCCI proteins in our cohort was most prominent in FTD-GRN when compared to FTD-MAPT. EWCE analysis showed enrichment for neurons, suggesting they might be the focal site of mitochondrial dysregulation.

In FTD-MAPT, protein signatures indicated affected processing and trafficking of RNA. Disturbances of RNA processing and nucleocytoplasmic transport (NCT) have repeatedly been reported in FTD in relation to TDP-43 [12, 22, 40, 51], FUS [40], and *C9ORF72* repeat expansion [21, 67]. Recently, for the first time, FTD-related *MAPT* mutations were linked to microtubule-mediated nuclear deformation and disruption of NCT in human iPSC-derived neurons [53]. By specifically selecting genetic cases in our cohort we were able to more prominently highlight the involvement of RNA processing and transport in FTD-MAPT. Comparison with AD supported the view that these processes are specifically associated with FTD-MAPT. Our results also illustrate that FTD-MAPT cases and FTD cases harbouring TDP-43 pathology (FTD-TDP) regardless of the presence of genetic mutations, might be more alike than previously expected.

In addition, ‘Axon’ and ‘Plasma membrane’ proteins showed enrichment for neurons and oligodendrocytes in FTD-MAPT. Interestingly, tau inclusions have been described in oligodendrocytes of patients with FTD-MAPT [16, 34], and studies with tau transgenic mice indicate that these inclusions disrupt axonal transport, leading to impairments in myelin and axon integrity [29]. Alternatively, tau accumulation in axons may indirectly impair oligodendrocyte function due to their functional interaction. Comparison of FTD-MAPT to AD highlighted involvement of the myelin sheath as highly distinctive, hinting at impaired axon-myelin interactions in FTD-MAPT. Perhaps the distinguishing factor in FTD-MAPT is indeed the presence of tau pathology of genetic origin in all cell types, versus the neuronal and extracellular tau pathology in (non-genetic) AD.

A potential limitation of proteomic studies using whole-tissue is the fact that protein abundances are dependent on expression changes in multiple cell types, and are consequently affected by changes in cell type ratios within the studied tissue. As neuronal loss is characteristic of neurodegeneration, changes in cell type ratios within the diseased brain are expected. However, our analysis suggested that detected protein expression differences are not simply caused by disease-induced cell loss or gain. Furthermore, to improve stratification of brain cell types involved in the FTD subtypes, we applied cell type enrichment analysis. This approach seems to adequately address the issue of mixed cell type populations, as we were able to demonstrate the involvement of different cell types within our whole-tissue data. Nonetheless, the results should be carefully interpreted, as our method of using scRNAseq data sets for the inference of cell types on a protein level has limitations as well. For instance, it is well known from literature that mRNA-to-protein correlations are only ~ 40% [17, 28, 36, 37, 55, 66, 72] and so mRNA specificity across cell types might not always reflect the cell type specificity at the protein level. In addition, a recent study has demonstrated a cell-type specific aging effect on the transcriptomic level [59], suggesting that age-related regulation of mRNA and protein levels might influence the cell type specificity ratios we infer from scRNAseq data sets. A benefit from our ‘enrichment’-based approach is that cell type inference is done using protein groups and not individual proteins, which reduces the possible effect of individual poorly-correlated or aging-sensitive proteins. Future (single) cell type-specific proteomics approaches [15, 44, 73] may further help disentangle the different causal or consequential processes for brain cell types in FTD.

## Conclusion

This study established the existence of distinct proteins, pathways, and cells affected within two genetic FTD subtypes, which might facilitate the development of specific cellular and/or animal FTD models, and the exploration of subtype-specific therapeutic targets. Moreover, proteomic studies of genetic FTD provide the framework for understanding both common neurodegenerative mechanisms and distinct processes underlying the genetic heterogeneity in FTD. Finally, it will be interesting to determine how sporadic FTD relates to the currently established common and subtype-specific protein signatures.

## Supplementary Information

**Additional file 1** Neuropathological characterization of FTD-GRN and FTD-MAPT cases included in the RiMOD-FTD cohort. FTD-GRN cases showed diffuse atrophy, most pronounced in the frontal lobes. The hippocampus was usually small and atrophic and showed hippocampal sclerosis in some cases. Microscopic examination revealed abnormal lamination and spongiosis of the second and third layers of frontal and temporal cortices, including insular and cingulate cortex, basal nuclei, and thalamus. Widespread pTDP-immunoreactivity in an S82VfsX174 mutation carrier demonstrates the presence of round or crescent cytoplasmic neuronal inclusions, intranuclear lentiform (“cat-eye”) inclusions (inset in B), and short dystrophic neurites with TDP-43 immunoreactivity in the affected frontal (A) and temporal (B) cortical regions, consistent with TDP subtype A. FTD-MAPT cases were characterized by profound symmetric atrophy of the anterior temporal lobe, usually extending to the parietal lobe. Frontal atrophy was often present, albeit slightly milder in some cases. Microscopically, prominent neuronal loss and gliosis was observed in the cerebral cortex, subcortical nuclei, amygdala, white matter, and brain stem. The extent of degeneration was comparable across cases, except for moderate degeneration in a single P301L carrier with a disease duration of 3 years, who died of sudden cardiac arrest. Tau-positive neuronal inclusions and neuropil threads and tangles were most abundant in regions with severe neuronal loss. Pick body-like inclusions were found in G272V cases (C, frontal cortex), whereas abundant AT8-immunoreactive ring-like neuronal inclusions and pre-tangles were observed in both neuronal and glial cells in P301L cases (D, temporal cortex). The single R406W mutation case showed many tangles and tau-positive neurons in the cortex, basal nuclei, and hippocampus. All scale bars shown are 20um).

**Additional file 2** High reproducibility in the SWATH proteomics experiment. Analysis of coefficient of variation (CoV) for protein abundances in technical replicates taken along in our SWATH proteomics analysis demonstrates high reproducibility in our experiment.

**Additional file 3** Lists of unique proteins detected and quantified within frontal and temporal cortical tissues for the RiMOD-FTD genetic subtypes. Proteins were selected using quality filtering on peptide level (*q* ≤10-3 in at least 50% of samples per group, i.e. NDC or FTD). For every protein, the raw fold change, raw *p*-value, multiple comparison corrected *q*-value, and effect size (d) are given for the statistical comparison between NDC and FTD subtype. In addition, columns are included which note whether a protein has passed statistical testing (either *p* < 0.05 or *q* < 0.05). Furthermore, the differential expression of several well-known neurodegeneration (ND)-related proteins in frontal cortical FTD-GRN *vs* NDC and temporal cortical FTD-MAPT *vs* NDC are highlighted. -; not detected within study cohort.

**Additional file 4** The majority of cell type-specific proteins shows expression differences within the range of NDC expression. Density plots for protein expression fold changes in FTD cases *vs* NDCs for proteins that are highly enriched for specific brain cell types or for the synapse demonstrate that the bulk of measured highly enriched proteins falls within the range of NDC protein variation, and that fold changes range from negative (lower expressed) to positive (higher expressed) values. (A) Fold change density plot for proteins highly enriched for astrocytes. (B) Fold change density plot for proteins highly enriched for endothelial cells. (C) Fold change density plot for proteins highly enriched for microglia. (D) Fold change density plot for proteins highly enriched for neuronal cell types. In this plot, proteins for excitatory and inhibitory neurons are taken together. (E) Fold change density plot for proteins highly enriched for oligodendrocytes. (F) Fold change density plot for proteins highly enriched for oligodendrocyte precursor cells. (G) Fold change density plot for proteins enriched for the pre- and postsynapse, as annotated by SynGO (see methods). Protein expression variation present in NDCs is depicted using dashed lines, which are set at two times the standard deviation for NDC *vs* NDC protein expression fold changes.

**Additional file 5** Lists of significant GO enrichment analysis results for proteins differentially expressed in frontal cortical FTD-GRN *vs* NDC and temporal cortical FTD-MAPT *vs* NDC. GO enrichment analysis was performed on proteins differentially expressed at *q* < 0.05, with proteins divided into lower and higher expressed proteins. For every GO term, the corresponding GO group is listed, as well as whether the GO term is considered to be a ‘Best Per Parent’ term.

**Additional file 6** SynGO analysis indicates generally affected synapses in FTD-GRN and FTD-MAPT. SynGO enrichment analysis on differentially expressed proteins shows synaptic proteins are related to a wide range of synaptic compartments and functions, indicating that synapses are more generally affected in both the FTD-GRN and FTD-MAPT subtype. SynGO analysis was performed on functional (BP) and location (CC) ontology terms. Statistical enrichment analysis was done using a one-sided Fisher exact test with a multiple testing correction using a 1% FDR. Sunburst plots are given both for ‘gene count per term’ and ‘enrichment value (-log10 *q*-value)’. (A,B) SynGO location analysis on lower expressed proteins in frontal cortical FTD-GRN. For this analysis, statistically significant enrichment is seen for several postsynaptic terms. (C,D) SynGO location analysis on higher expressed proteins in frontal cortical FTD-GRN. (E,F) SynGO location analysis on lower expressed proteins in temporal cortical FTD-MAPT. (G,H) SynGO location analysis on higher expressed proteins in temporal cortical FTD-MAPT. (I,J) SynGO functional analysis on lower expressed proteins in frontal cortical FTD-GRN. For this analysis, statistically significant enrichment is seen related to structural synaptic organization. (K,L) SynGO functional analysis on higher expressed proteins in frontal cortical FTD-GRN. (M,N) SynGO functional analysis on lower expressed proteins in temporal cortical FTD-MAPT. For this analysis, statistically significant enrichment is seen for presynaptic and general synaptic signalling terms. (O,P) SynGO functional analysis on higher expressed proteins in temporal cortical FTD-MAPT. BDNF, brain-derived neurotrophic factor, DCV; dense core vesicle, ECM; extracellular matrix, IC; ion channel, IF; intermediate filament, NT; neurotransmitter, NTR; neurotransmitter receptor, PSD; postsynaptic density, SV; synaptic vesicle

**Additional file 7** Lists of SynGO enrichment analysis results for proteins differentially expressed in frontal cortical FTD-GRN vs NDC and temporal cortical FTD-MAPT *vs* NDC. SynGO enrichment analysis was performed on proteins differentially expressed at *q* < 0.05, with proteins divided into lower and higher expressed proteins. For every SynGO term, the proteins within that term that were measured in the different FTD subtypes are given, as well the raw and multiple testing corrected *p*-value for the enrichment analysis.

**Additional file 8** Overview of MitoCarta-based analysis for proteins differentially expressed in frontal cortical FTD-GRN *vs* NDC. MitoCarta-based analysis was performed on proteins differentially expressed at *q* < 0.05. Results include MitoCarta protein annotations, results from the PANTHER overrepresentation analysis on the MitoCarta-annotated proteins, and an overview of those MitoCarta proteins involved in the respiratory chain complexes I-III

**Additional file 9** Comparison of protein effect sizes related to immune processes, metabolism, and mitochondria demonstrates that these are most affected in FTD-GRN. (A) Effect size plot comparing differentially expressed proteins from the ‘Metabolism’ GO group in FTD-GRN with FTD-MAPT. (B) Effect size plot comparing differentially expressed proteins from the ‘Immune’ GO group in FTD-GRN with FTD-MAPT. (C) Effect size plot comparing differentially expressed proteins from the ‘Mitochondria’ GO group in FTD-GRN with FTD-MAPT. Comparisons shows that, though (a portion of) proteins are affected in the other FTD subtype as well, these processes seem to be most affected in FTD-GRN. d; statistical effect size SAM analysis.

**Additional file 10** Comparison of protein effect sizes related to RNA processing, axons, ion transport, and the plasma membrane demonstrates that these are most affected in FTD-MAPT. (A) Effect size plot comparing differentially expressed proteins from the ‘RNA processing’ GO group in FTD-MAPT with FTD-GRN. (B) Effect size plot comparing differentially expressed proteins from the ‘Axon’ GO group in FTD-MAPT with FTD-GRN. (C) Effect size plot comparing differentially expressed proteins from the ‘Ion transport’ GO group in FTD-MAPT with FTD-GRN. (D) Effect size plot comparing differentially expressed proteins from the ‘Plasma membrane’ GO group in FTD-MAPT with FTD-GRN. Comparisons shows that, though (a portion of) proteins are affected in the other FTD subtype as well, these processes seem to be most affected in FTD-MAPT. d; statistical effect size SAM analysis.

**Additional file 11** Validation of the distinct involvement of proteins in FTD subtypes using immunoblotting. (A) Analysis of several mitochondrial respiratory chain subunits in frontal cortical tissues. Annotated immunoblots used for the analysis of OXPHOS antibody signals (chemi channel) are shown. Average protein signals are quantified and shown in dot plots. Differences in protein expression levels were analysed per group comparison using a Student’s t-test (proteins I-IV) or a Welch’s t-test (protein V). Respiratory complex proteins I-IV show a lower expression in FTD-GRN while remaining virtually unchanged in FTD-MAPT. Fold changes for FTD-GRN vs NDC are 0.77, 0.67, 0.75, and 0.77, respectively, though differences are not statistically significant. Respiratory complex protein V shows a higher expression in both FTD-GRN (1.20x) and FTD-MAPT (1.55x) compared to NDC, with a statistically significant difference for FTD-MAPT (*p* = 0.0433). All five independent samples show comparable expression levels to those of the original cohort. (B) Analysis of PLP1 in temporal cortical tissues. Annotated immunoblots used for the analysis of PLP1 antibody signals (chemi channel) are shown. Average protein signals are quantified and shown in dot plots. Differences in protein expression levels were analysed per group comparison using a Student’s t-test. PLP1 shows a lower expression in both FTD-GRN (0.44x) and FTD-MAPT (0.39x) compared to NDC, with a strong trend for FTD-MAPT (*p* = 0.0595). All five independent samples show comparable expression levels to those of the original cohort. Numbers represent apparent molecular weights in kDa. Letters represent sample annotations. Protein signal values are corrected for gel loading differences and are normalized to NDC samples. G; FTD-GRN sample, GN; FTD-GRN sample from the independent cohort, M; FTD-MAPT sample, MN; FTD-MAPT sample from the independent cohort, N; non-demented control sample.

**Additional file 12** Raw images of OXPHOS and PLP1 immunoblotting experiments. (A) Total protein load for each sample on the gels used in the OXPHOS immunoblotting experiments (Bio-Rad gel images). Specific antibody signal values on the corresponding blots are corrected for gel loading differences using these images. (B) Whole immunoblot images used for the analysis of several mitochondrial respiratory chain subunits in frontal cortical tissues. Signals from the chemi and 700 nm channels are shown. (C) Total protein load for each sample on the gels used in the PLP1 immunoblotting experiments (Bio-Rad gel images). Specific antibody signal values on the corresponding blots are corrected for gel loading differences using these images. (D) Whole immunoblot images used for the analysis of myelin-associated protein PLP1 in temporal cortical tissues. Signals from the chemi and 700 nm channels are shown.

**Additional file 13** Overview of unique proteins detected and quantified within temporal cortical tissues for AD and unique proteins detected and quantified within temporal cortical tissues for both AD and the FTD-MAPT subtype. Proteins were selected using quality filtering on peptide level (*q* ≤10-3 in at least 50% of samples per group, i.e. NDC or AD). For every protein, the raw fold change and raw *p*-value are given for the statistical comparison between NDC and AD, and NDC and FTD-MAPT. In addition, columns are included which note whether a protein has passed statistical multiple testing comparison (*q* < 0.05) and whether differential protein expression is in a similar direction in AD and FTD-MAPT.

**Additional file 14** Lists of significant GO enrichment analysis results for proteins differentially expressed in both temporal cortical FTD-MAPT *vs* NDC and AD *vs* NDC. GO enrichment analysis was performed for proteins differentially expressed at *q* < 0.05 in both temporal cortical AD and FTD-MAPT *vs* NDC, and for proteins that are only differentially expressed (*q* < 0.05) in temporal cortical FTD-MAPT *vs* NDC. Differentially expressed proteins are divided into lower and higher expressed proteins. For every GO term, the corresponding GO group is listed, as well as whether the GO term is considered to be a ‘Best Per Parent’ term.

**Additional file 15** Lists of SynGO enrichment analysis results for proteins differentially expressed in both temporal cortical FTD-MAPT *vs* NDC and AD *vs* NDC. SynGO enrichment analysis was performed for proteins differentially expressed at *q* < 0.05, with proteins divided into lower and higher expressed proteins. For every SynGO term, the proteins within that term that were measured in the different FTD subtypes are given, as well the raw and multiple testing corrected *p*-value for the enrichment analysis.

## Data Availability

The mass spectrometry proteomics data of the FTD cohort have been deposited to the ProteomeXchange Consortium via the PRIDE [54] partner repository with the dataset identifier PXD031419. The mass spectrometry proteomics data of the AD cohort are not publicly available yet as they are part of a larger data set that will be published separately but are available from the authors upon reasonable request. All data from downstream bioinformatics analysis for both cohorts and from immunoblotting experiments are included in this article and its additional files.

## Abbreviations

AD: Alzheimer’s disease
ALS: Amyotrophic lateral sclerosis
BBB: Blood-brain-barrier
BP: Biological process
CC: Cellular component
DDA: Data-dependent acquisition
DIA: Data-independent acquisition
EWCE: Expression-weighted cell type enrichment
FDR: False discovery rate
FTD: Frontotemporal dementia
FTD-C9: *C9ORF72*-mediated genetic subtype of FTD
FTD-GRN: *GRN*-mediated genetic subtype of FTD
FTD-MAPT: *MAPT*-mediated genetic subtype of FTD
FTD-TDP: FTD subtype characterized by TDP-43 pathology
GO: Gene ontology
LC–MS/MS: Liquid chromatography with tandem mass spectrometry
MF: Molecular function
NCT: Nucleocytoplasmic transport
NDC: Non-demented control
OPC: Oligodendrocyte precursor cell
RCCI: Respiratory chain complex I
SV: Synaptic vesicle
