# Supplementary figures and images for: Distinct cell type-specific protein signatures in GRN and MAPT genetic subtypes of frontotemporal dementia
Source: Acta Neuropathol Commun. 2022 Jul 7;10:100. doi: 10.1186/s40478-022-01387-8 (PMC9261008; doi:10.1186/s40478-022-01387-8)

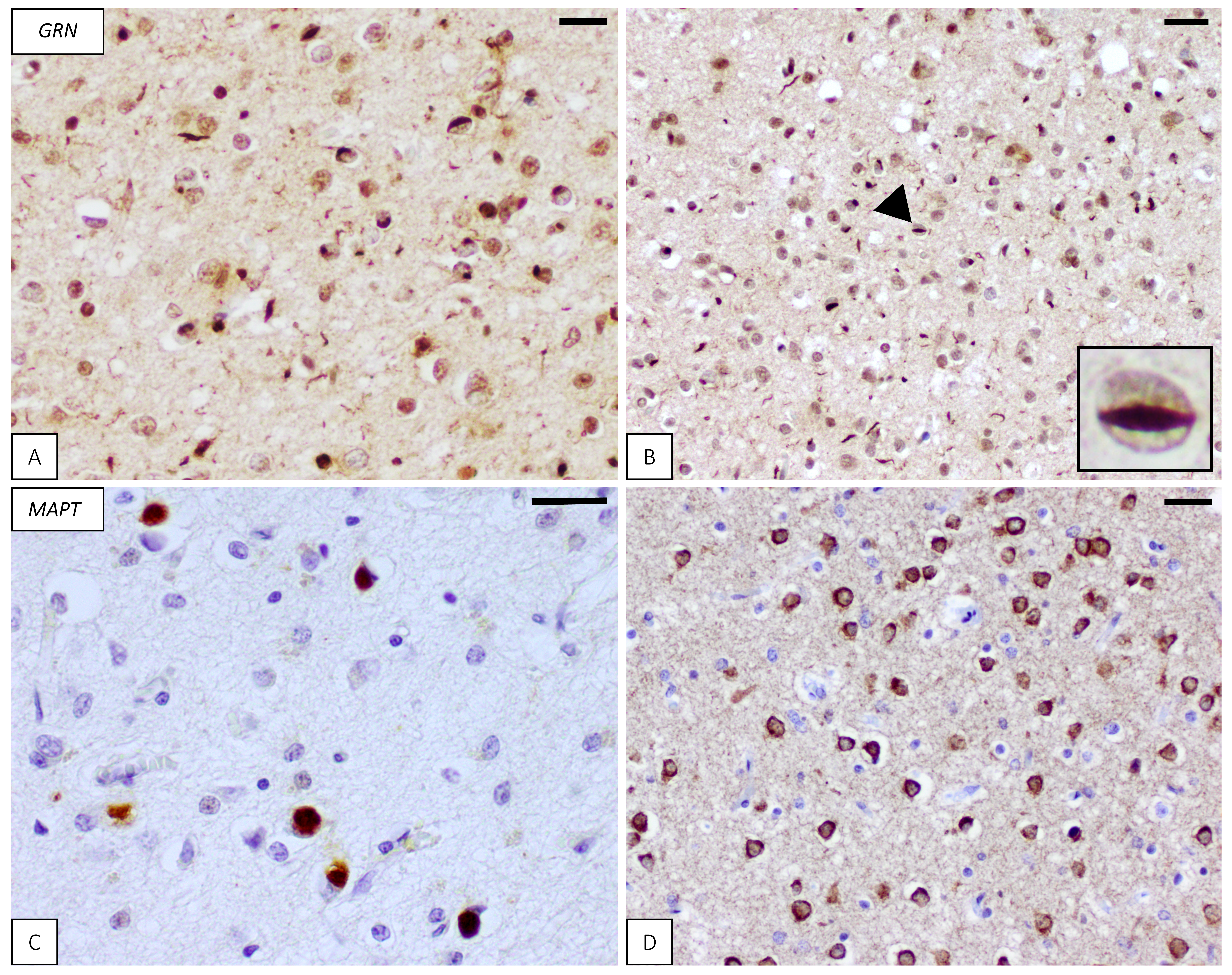

Supplement: Supplementary file 1 — Additional file 1 Neuropathological characterization of FTD-GRN and FTD-MAPT cases included in the RiMOD-FTD cohort. FTD-GRN cases showed diffuse atrophy, most pronounced in the frontal lobes. The hippocampus was usually small and atrophic and showed hippocampal sclerosis in some cases. Microscopic examination revealed abnormal lamination and spongiosis of the second and third layers of frontal and temporal cortices, including insular and cingulate cortex, basal nuclei, and thalamus. Widespread pTDP-immunoreactivity in an S82VfsX174 mutation carrier demonstrates the presence of round or crescent cytoplasmic neuronal inclusions, intranuclear lentiform (“cat-eye”) inclusions (inset in B), and short dystrophic neurites with TDP-43 immunoreactivity in the affected frontal (A) and temporal (B) cortical regions, consistent with TDP subtype A. FTD-MAPT cases were characterized by profound symmetric atrophy of the anterior temporal lobe, usually extending to the parietal lobe. Frontal atrophy was often present, albeit slightly milder in some cases. Microscopically, prominent neuronal loss and gliosis was observed in the cerebral cortex, subcortical nuclei, amygdala, white matter, and brain stem. The extent of degeneration was comparable across cases, except for moderate degeneration in a single P301L carrier with a disease duration of 3 years, who died of sudden cardiac arrest. Tau-positive neuronal inclusions and neuropil threads and tangles were most abundant in regions with severe neuronal loss. Pick body-like inclusions were found in G272V cases (C, frontal cortex), whereas abundant AT8-immunoreactive ring-like neuronal inclusions and pre-tangles were observed in both neuronal and glial cells in P301L cases (D, temporal cortex). The single R406W mutation case showed many tangles and tau-positive neurons in the cortex, basal nuclei, and hippocampus. All scale bars shown are 20um). [file 40478_2022_1387_MOESM1_ESM.tif]

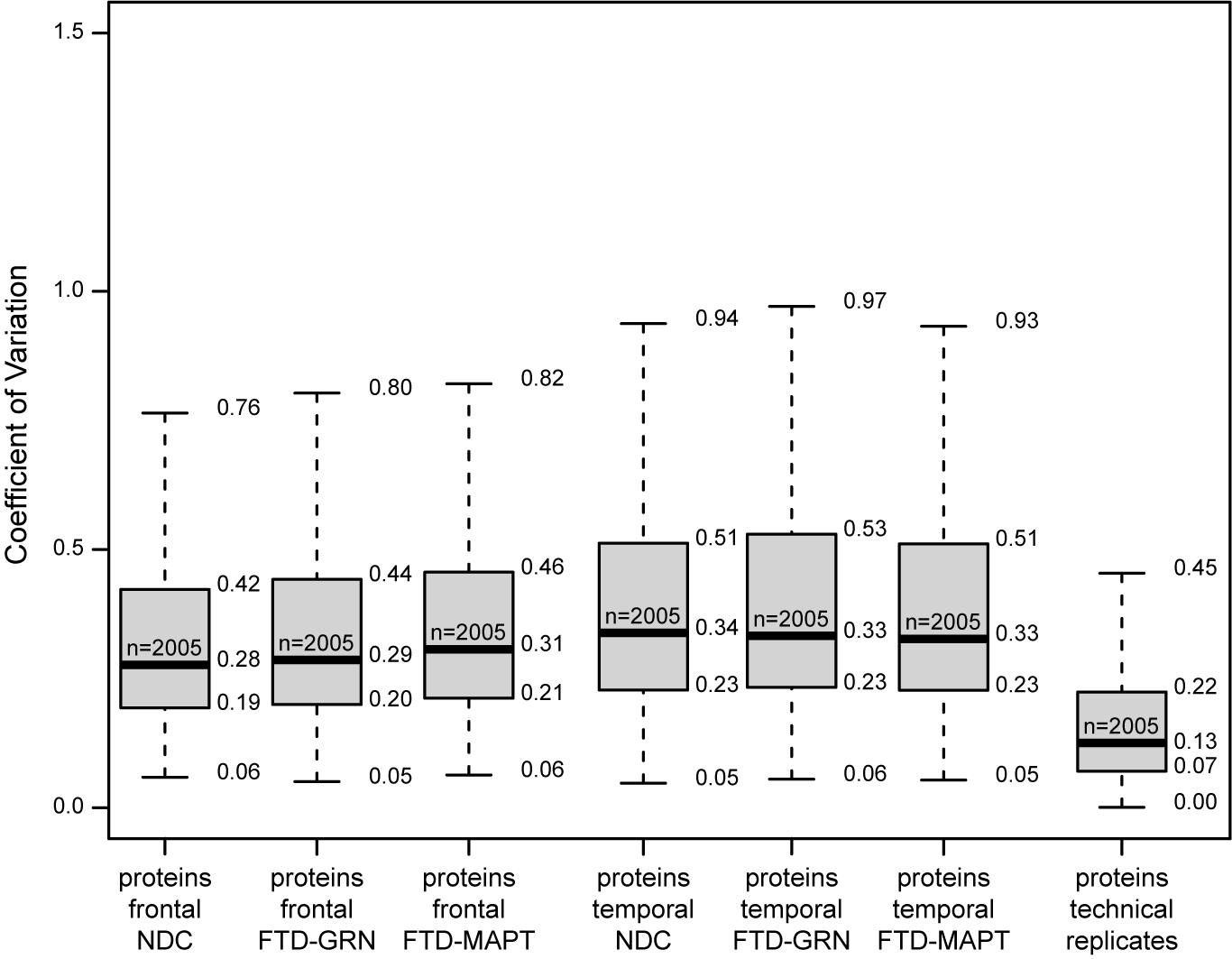

Supplement: Supplementary file 2 — Additional file 2 High reproducibility in the SWATH proteomics experiment. Analysis of coefficient of variation (CoV) for protein abundances in technical replicates taken along in our SWATH proteomics analysis demonstrates high reproducibility in our experiment. [file 40478_2022_1387_MOESM2_ESM.tif]

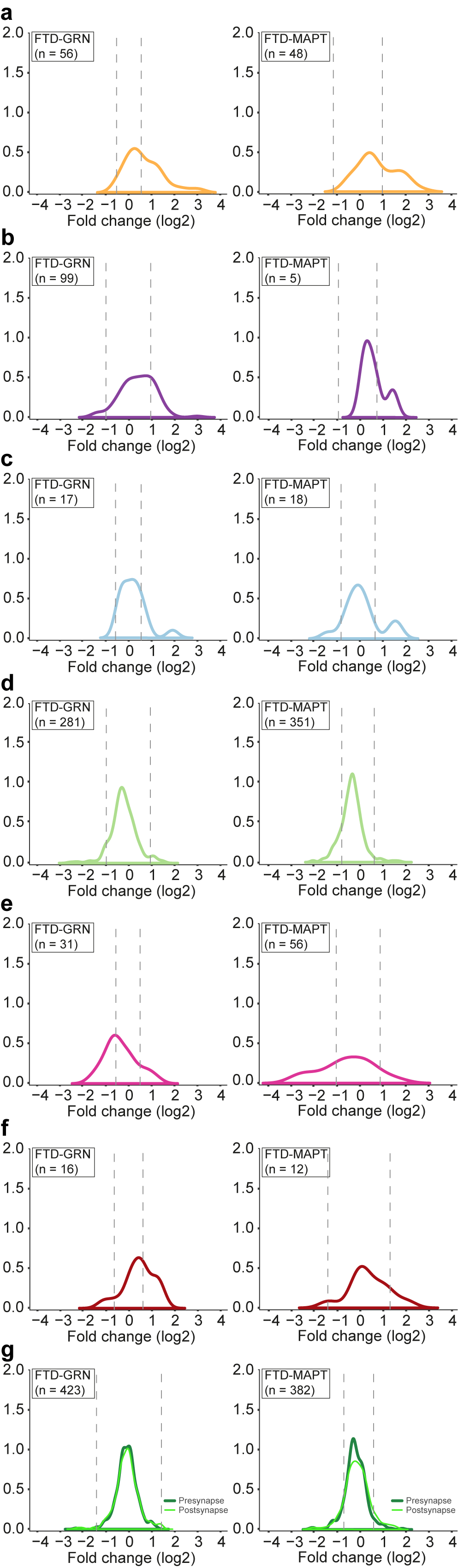

Supplement: Supplementary file 4 — Additional file 4 The majority of cell type-specific proteins shows expression differences within the range of NDC expression. Density plots for protein expression fold changes in FTD cases vs NDCs for proteins that are highly enriched for specific brain cell types or for the synapse demonstrate that the bulk of measured highly enriched proteins falls within the range of NDC protein variation, and that fold changes range from negative (lower expressed) to positive (higher expressed) values. (A) Fold change density plot for proteins highly enriched for astrocytes. (B) Fold change density plot for proteins highly enriched for endothelial cells. (C) Fold change density plot for proteins highly enriched for microglia. (D) Fold change density plot for proteins highly enriched for neuronal cell types. In this plot, proteins for excitatory and inhibitory neurons are taken together. (E) Fold change density plot for proteins highly enriched for oligodendrocytes. (F) Fold change density plot for proteins highly enriched for oligodendrocyte precursor cells. (G) Fold change density plot for proteins enriched for the pre- and postsynapse, as annotated by SynGO (see methods). Protein expression variation present in NDCs is depicted using dashed lines, which are set at two times the standard deviation for NDC vs NDC protein expression fold changes. [file 40478_2022_1387_MOESM4_ESM.tif]

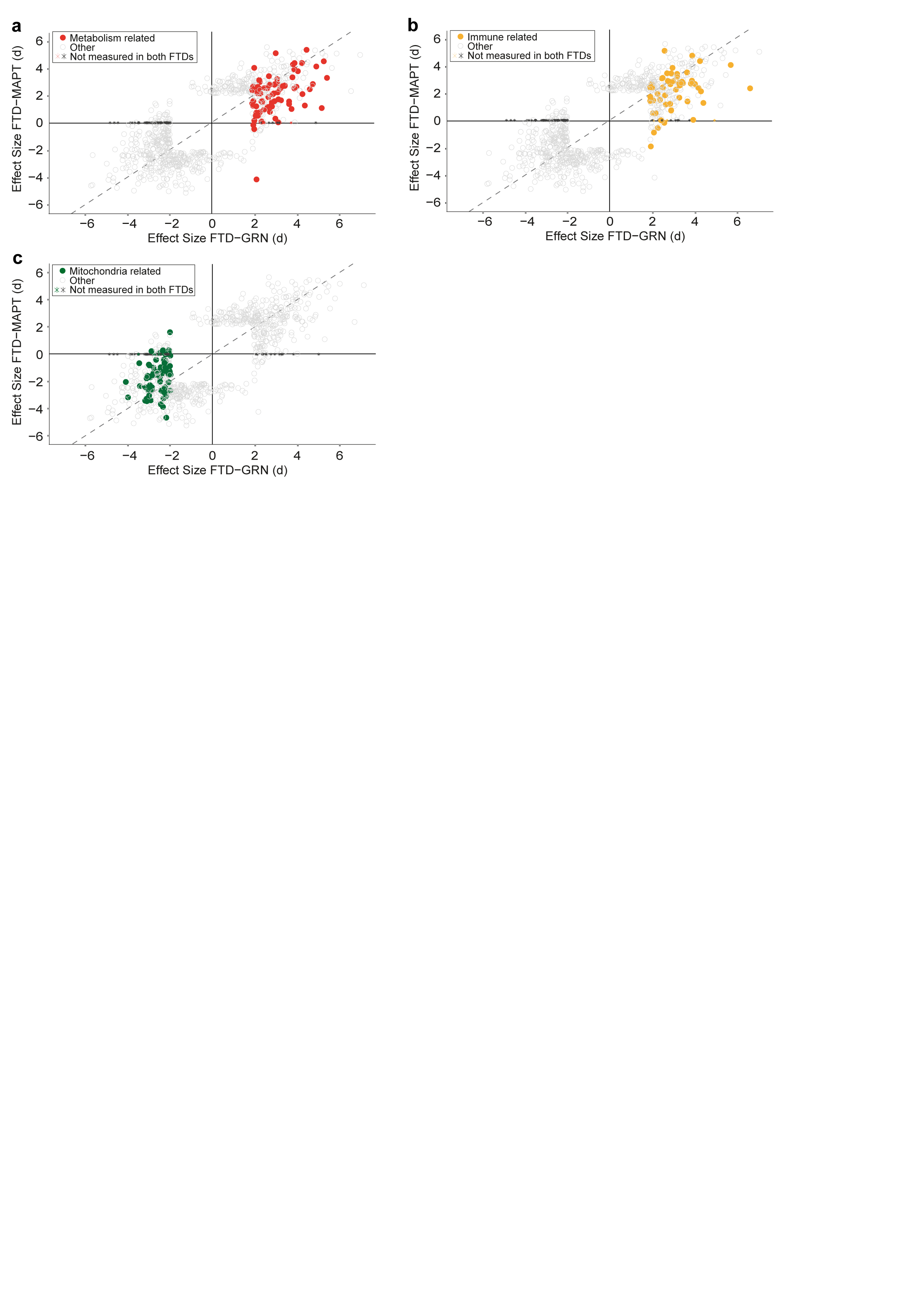

Supplement: Supplementary file 9 — Additional file 9 Comparison of protein effect sizes related to immune processes, metabolism, and mitochondria demonstrates that these are most affected in FTD-GRN. (A) Effect size plot comparing differentially expressed proteins from the ‘Metabolism’ GO group in FTD-GRN with FTD-MAPT. (B) Effect size plot comparing differentially expressed proteins from the ‘Immune’ GO group in FTD-GRN with FTD-MAPT. (C) Effect size plot comparing differentially expressed proteins from the ‘Mitochondria’ GO group in FTD-GRN with FTD-MAPT. Comparisons shows that, though (a portion of) proteins are affected in the other FTD subtype as well, these processes seem to be most affected in FTD-GRN. d; statistical effect size SAM analysis. [file 40478_2022_1387_MOESM9_ESM.tif]

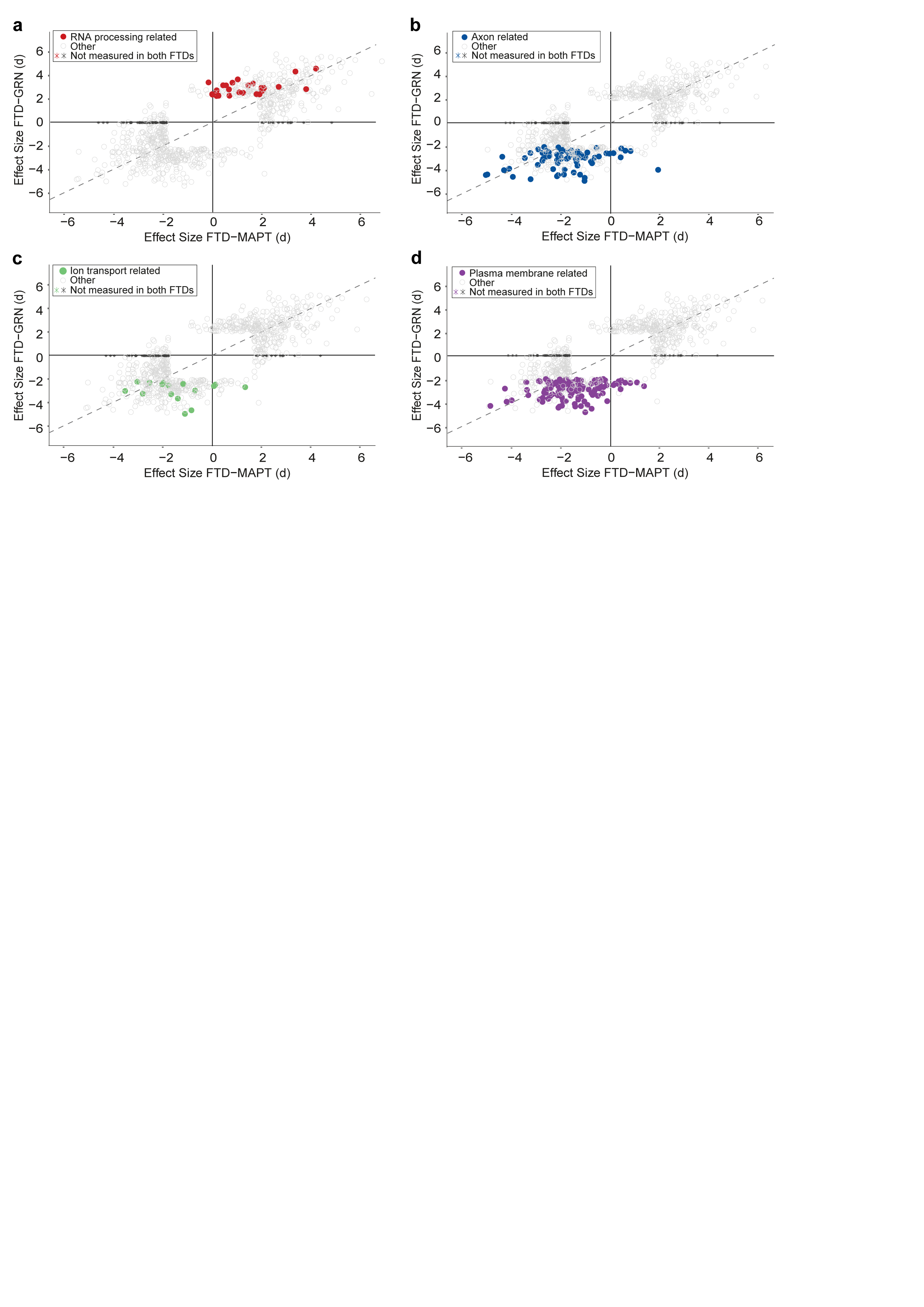

Supplement: Supplementary file 10 — Additional file 10 Comparison of protein effect sizes related to RNA processing, axons, ion transport, and the plasma membrane demonstrates that these are most affected in FTD-MAPT. (A) Effect size plot comparing differentially expressed proteins from the ‘RNA processing’ GO group in FTD-MAPT with FTD-GRN. (B) Effect size plot comparing differentially expressed proteins from the ‘Axon’ GO group in FTD-MAPT with FTD-GRN. (C) Effect size plot comparing differentially expressed proteins from the ‘Ion transport’ GO group in FTD-MAPT with FTD-GRN. (D) Effect size plot comparing differentially expressed proteins from the ‘Plasma membrane’ GO group in FTD-MAPT with FTD-GRN. Comparisons shows that, though (a portion of) proteins are affected in the other FTD subtype as well, these processes seem to be most affected in FTD-MAPT. d; statistical effect size SAM analysis. [file 40478_2022_1387_MOESM10_ESM.tif]

**A**

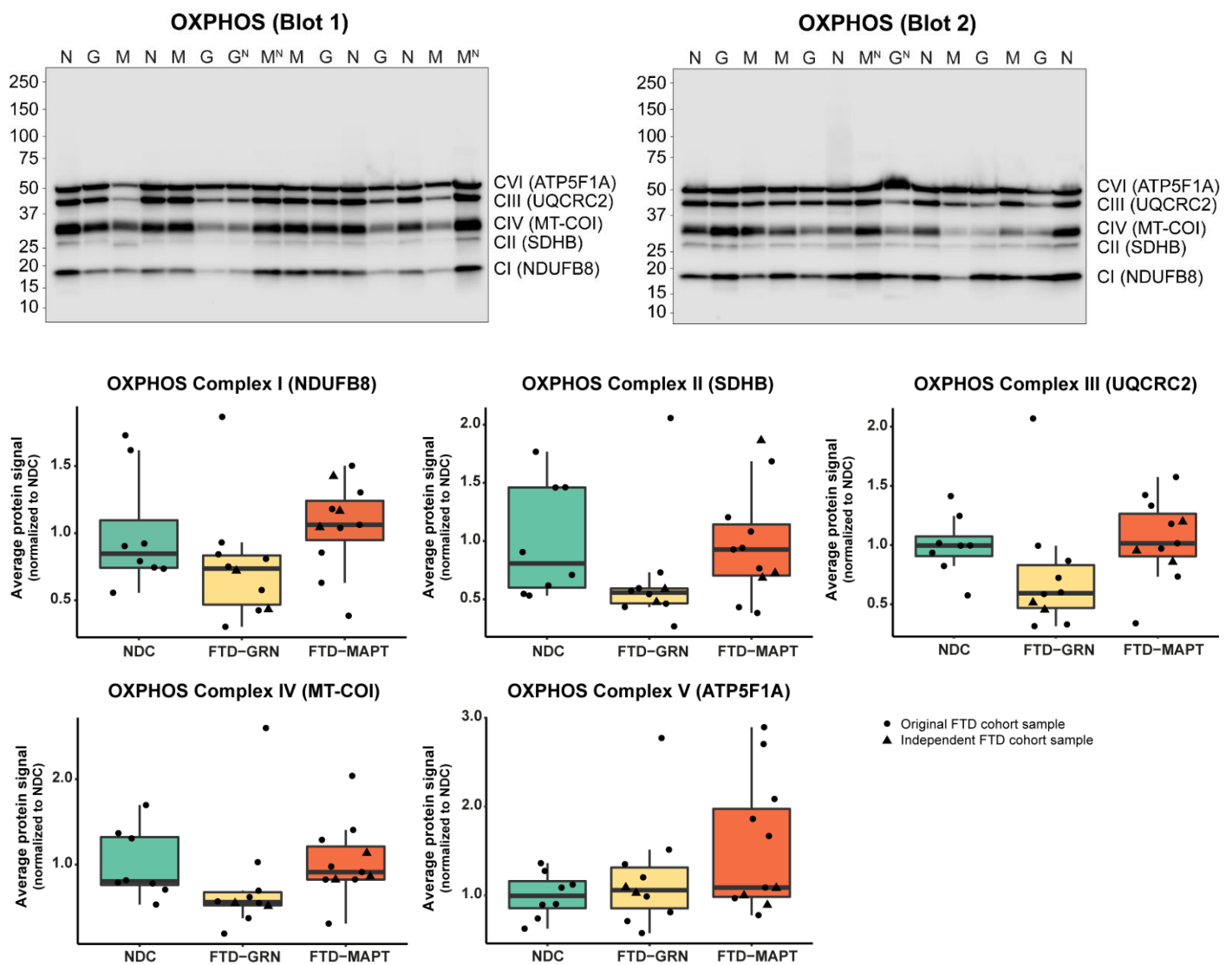

**B**

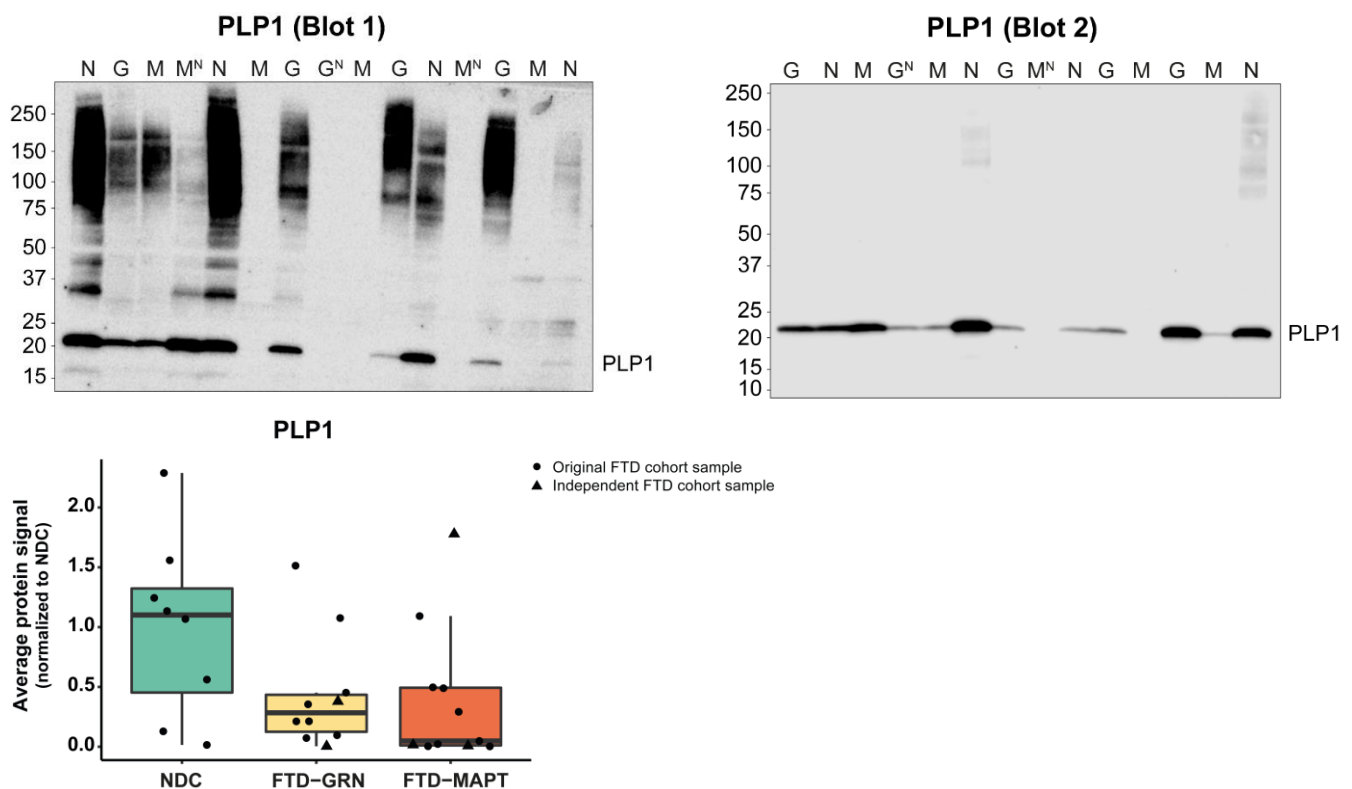

Supplement: Supplementary file 11 — Additional file 11 Validation of the distinct involvement of proteins in FTD subtypes using immunoblotting. (A) Analysis of several mitochondrial respiratory chain subunits in frontal cortical tissues. Annotated immunoblots used for the analysis of OXPHOS antibody signals (chemi channel) are shown. Average protein signals are quantified and shown in dot plots. Differences in protein expression levels were analysed per group comparison using a Student’s t-test (proteins I-IV) or a Welch’s t-test (protein V). Respiratory complex proteins I-IV show a lower expression in FTD-GRN while remaining virtually unchanged in FTD-MAPT. Fold changes for FTD-GRN vs NDC are 0.77, 0.67, 0.75, and 0.77, respectively, though differences are not statistically significant. Respiratory complex protein V shows a higher expression in both FTD-GRN (1.20x) and FTD-MAPT (1.55x) compared to NDC, with a statistically significant difference for FTD-MAPT (p = 0.0433). All five independent samples show comparable expression levels to those of the original cohort. (B) Analysis of PLP1 in temporal cortical tissues. Annotated immunoblots used for the analysis of PLP1 antibody signals (chemi channel) are shown. Average protein signals are quantified and shown in dot plots. Differences in protein expression levels were analysed per group comparison using a Student’s t-test. PLP1 shows a lower expression in both FTD-GRN (0.44x) and FTD-MAPT (0.39x) compared to NDC, with a strong trend for FTD-MAPT (p = 0.0595). All five independent samples show comparable expression levels to those of the original cohort. Numbers represent apparent molecular weights in kDa. Letters represent sample annotations. Protein signal values are corrected for gel loading differences and are normalized to NDC samples. G; FTD-GRN sample, GN; FTD-GRN sample from the independent cohort, M; FTD-MAPT sample, MN; FTD-MAPT sample from the independent cohort, N; non-demented control sample. [file 40478_2022_1387_MOESM11_ESM.pdf]

**A**

OXPHOS (Gel 1)

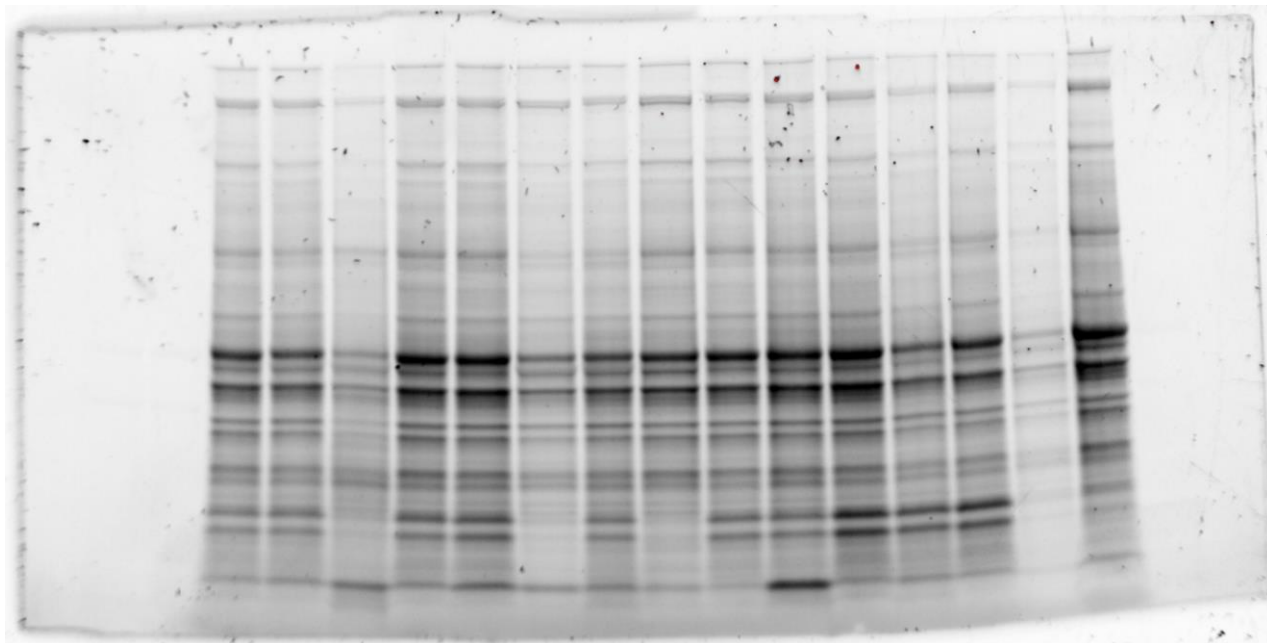

OXPHOS (Gel 2)

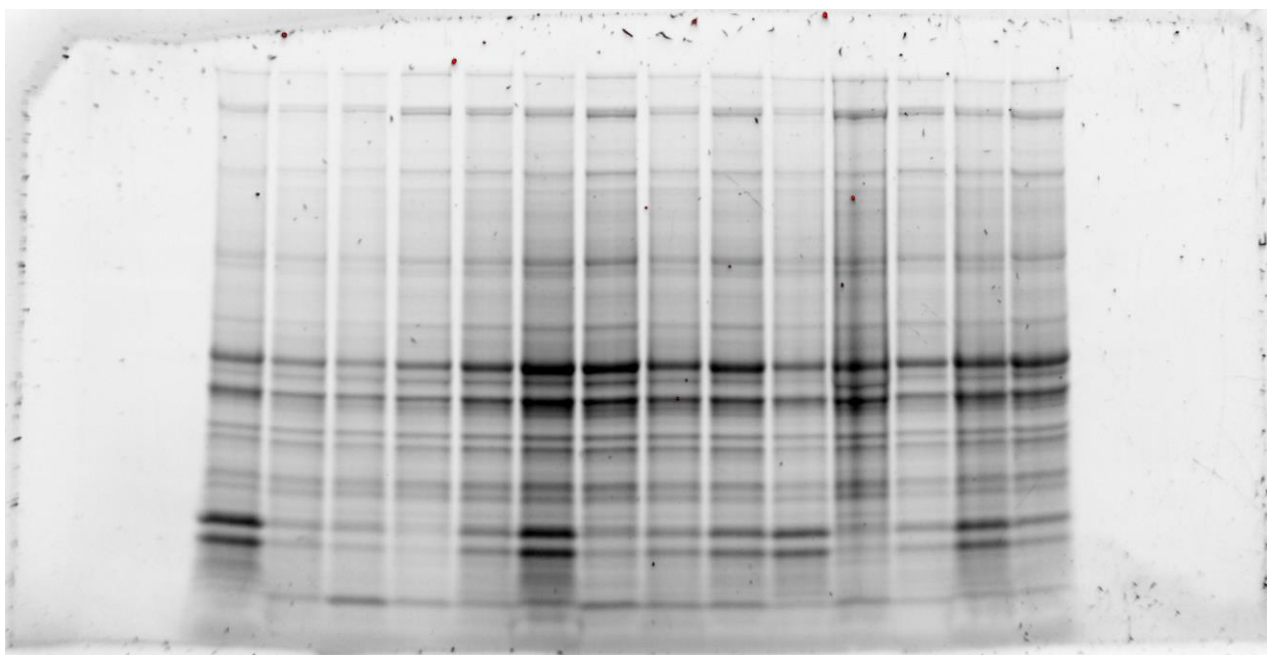

**B**

OXPHOS (Blot 1)

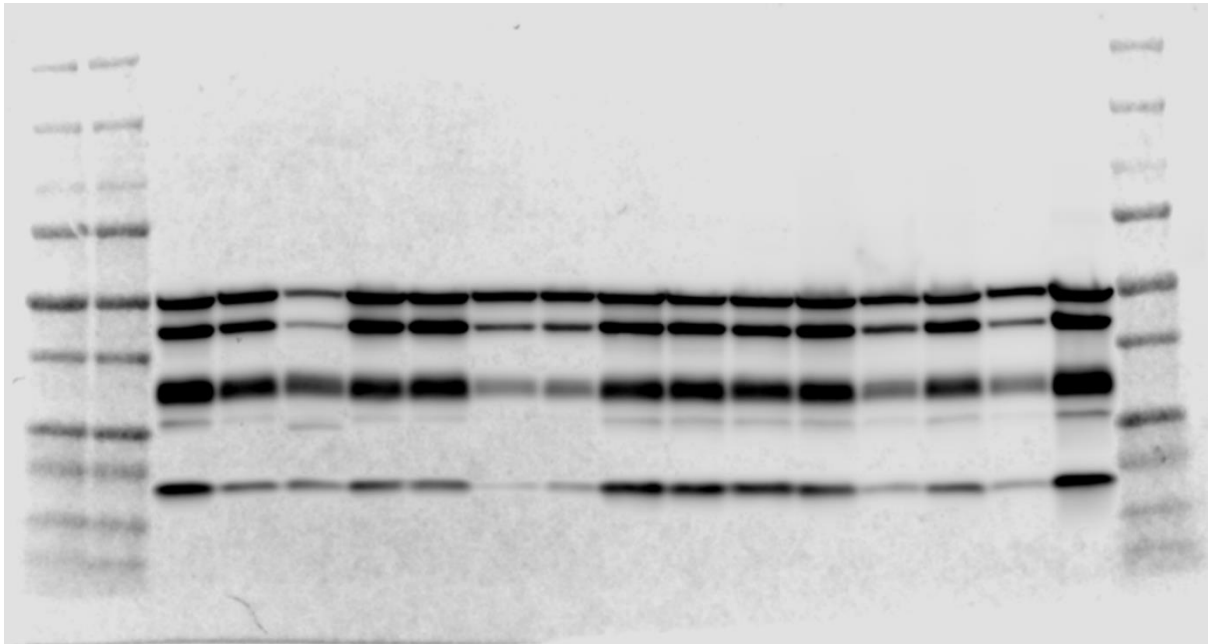

OXPHOS (Blot 2)

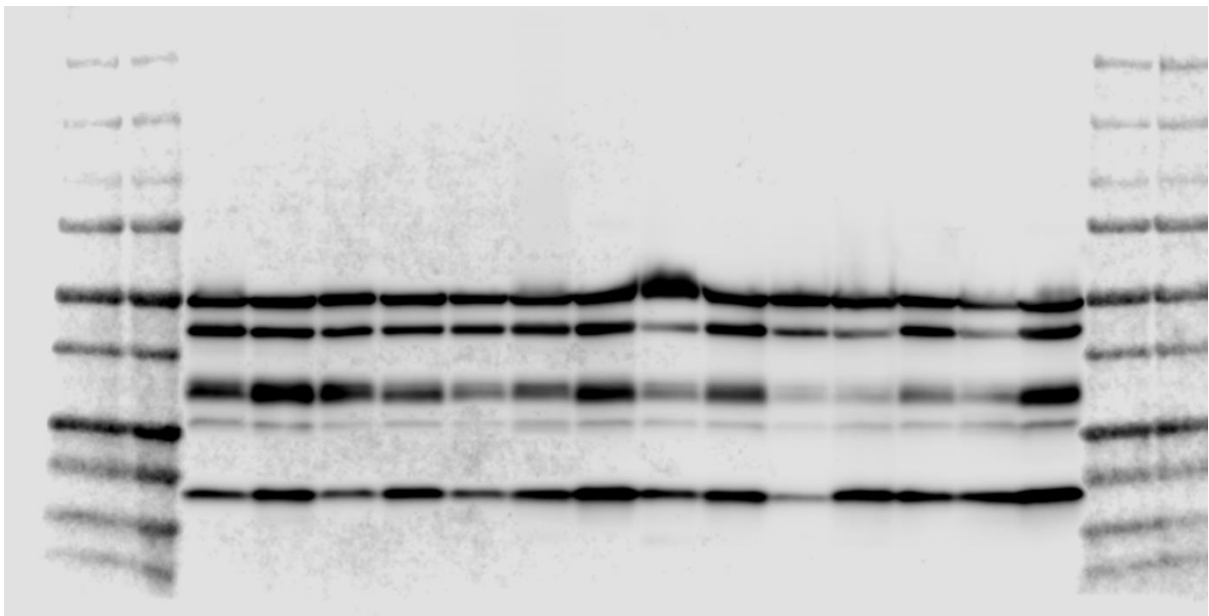

**C**

PLP1 (Gel 1)

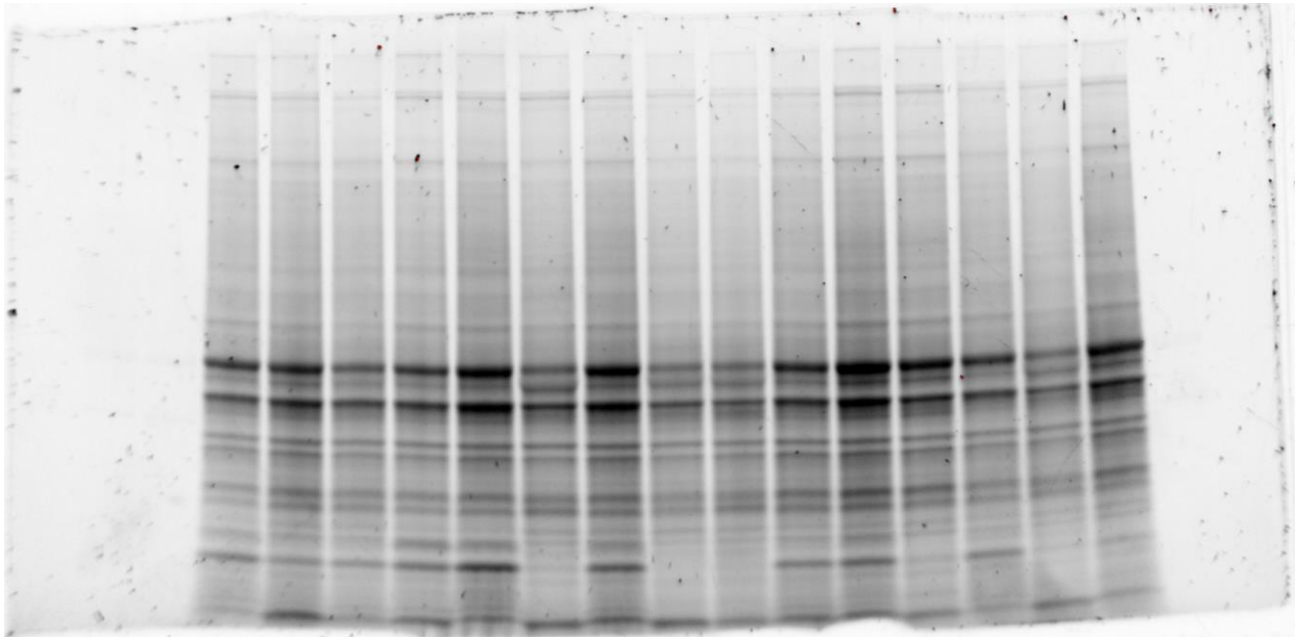

PLP1 (Gel 2)

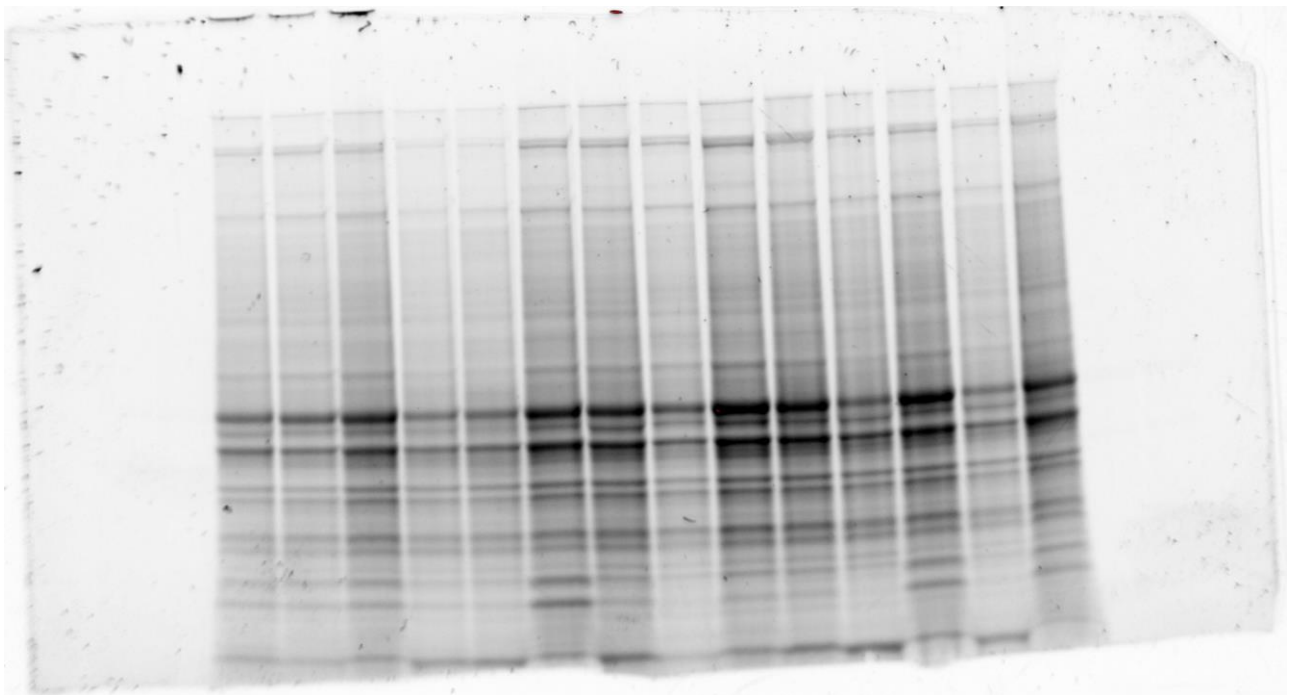

**D**

PLP1 (Blot 1)

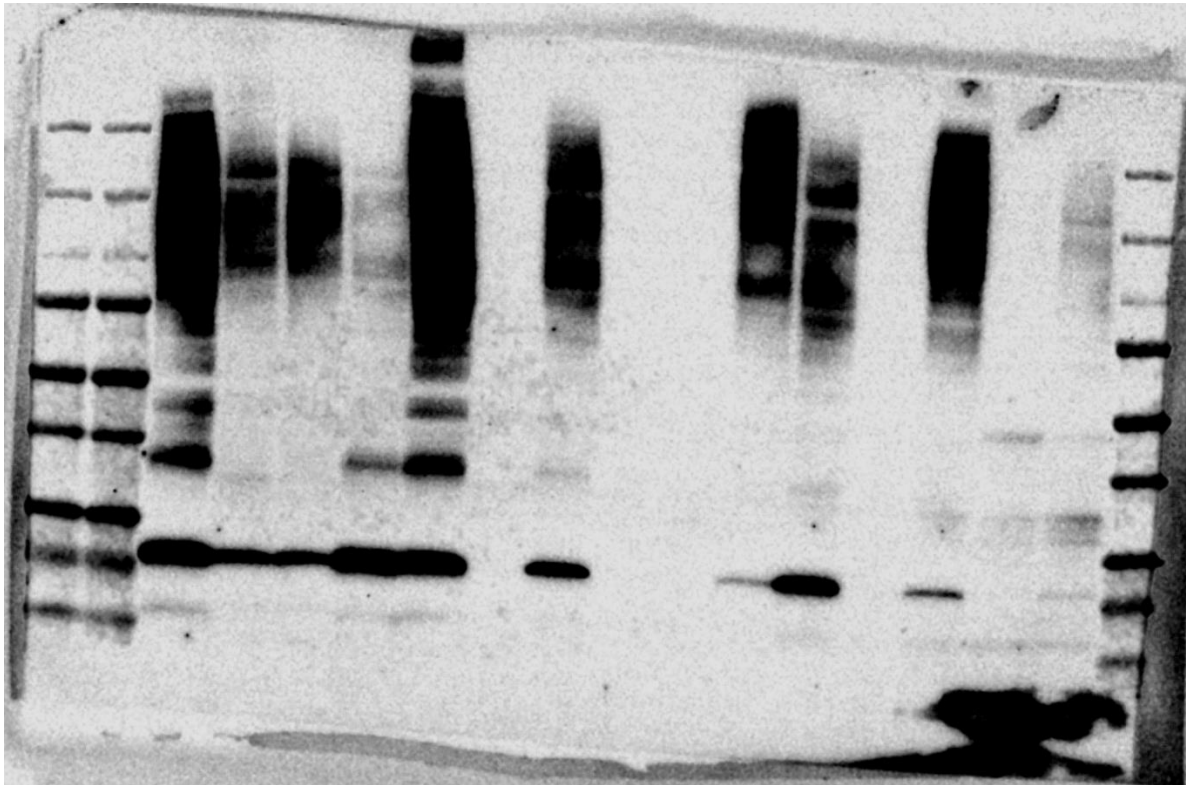

PLP1 (Blot 2)

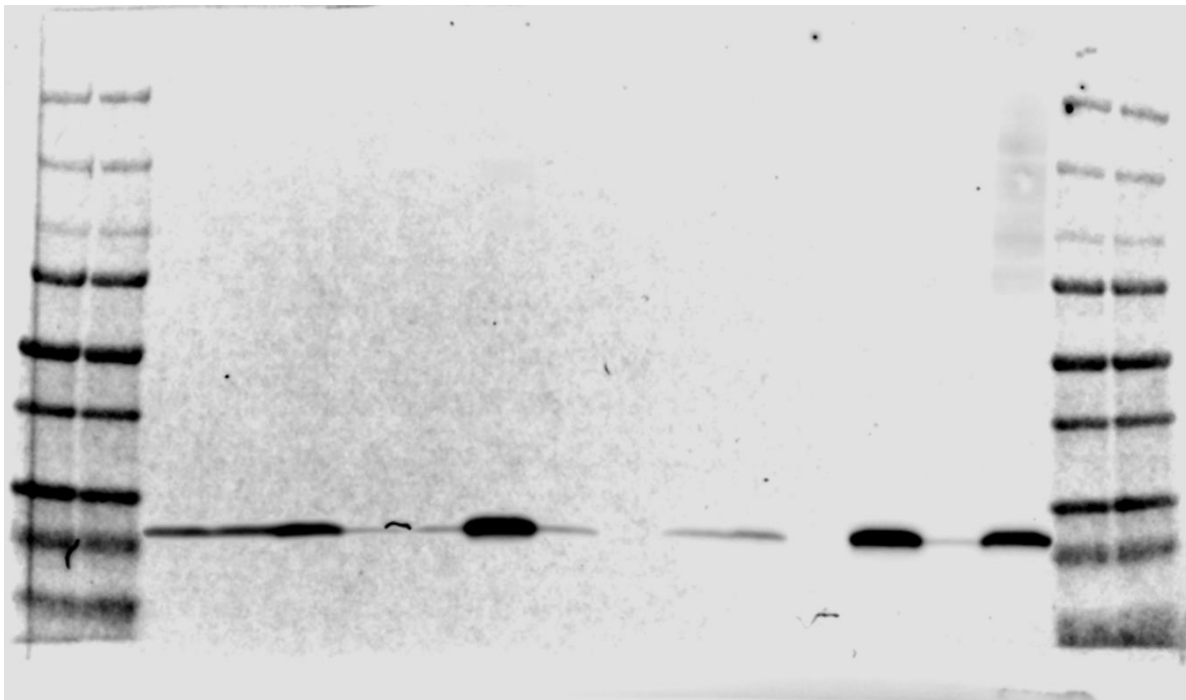

Supplement: Supplementary file 12 — Additional file 12 Raw images of OXPHOS and PLP1 immunoblotting experiments. (A) Total protein load for each sample on the gels used in the OXPHOS immunoblotting experiments (Bio-Rad gel images). Specific antibody signal values on the corresponding blots are corrected for gel loading differences using these images. (B) Whole immunoblot images used for the analysis of several mitochondrial respiratory chain subunits in frontal cortical tissues. Signals from the chemi and 700 nm channels are shown. (C) Total protein load for each sample on the gels used in the PLP1 immunoblotting experiments (Bio-Rad gel images). Specific antibody signal values on the corresponding blots are corrected for gel loading differences using these images. (D) Whole immunoblot images used for the analysis of myelin-associated protein PLP1 in temporal cortical tissues. Signals from the chemi and 700 nm channels are shown. [file 40478_2022_1387_MOESM12_ESM.pdf]
