# Supplementary material for: Distinct cell type-specific protein signatures in GRN and MAPT genetic subtypes of frontotemporal dementia
Source: Acta Neuropathol Commun. 2022 Jul 7;10:100. doi: 10.1186/s40478-022-01387-8 (PMC9261008; doi:10.1186/s40478-022-01387-8)
Supplement: Supplementary file 6 — Additional file 6 SynGO analysis indicates generally affected synapses in FTD-GRN and FTD-MAPT. SynGO enrichment analysis on differentially expressed proteins shows synaptic proteins are related to a wide range of synaptic compartments and functions, indicating that synapses are more generally affected in both the FTD-GRN and FTD-MAPT subtype. SynGO analysis was performed on functional (BP) and location (CC) ontology terms. Statistical enrichment analysis was done using a one-sided Fisher exact test with a multiple testing correction using a 1% FDR. Sunburst plots are given both for ‘gene count per term’ and ‘enrichment value (-log10 q-value)’. (A,B) SynGO location analysis on lower expressed proteins in frontal cortical FTD-GRN. For this analysis, statistically significant enrichment is seen for several postsynaptic terms. (C,D) SynGO location analysis on higher expressed proteins in frontal cortical FTD-GRN. (E,F) SynGO location analysis on lower expressed proteins in temporal cortical FTD-MAPT. (G,H) SynGO location analysis on higher expressed proteins in temporal cortical FTD-MAPT. (I,J) SynGO functional analysis on lower expressed proteins in frontal cortical FTD-GRN. For this analysis, statistically significant enrichment is seen related to structural synaptic organization. (K,L) SynGO functional analysis on higher expressed proteins in frontal cortical FTD-GRN. (M,N) SynGO functional analysis on lower expressed proteins in temporal cortical FTD-MAPT. For this analysis, statistically significant enrichment is seen for presynaptic and general synaptic signalling terms. (O,P) SynGO functional analysis on higher expressed proteins in temporal cortical FTD-MAPT. BDNF, brain-derived neurotrophic factor, DCV; dense core vesicle, ECM; extracellular matrix, IC; ion channel, IF; intermediate filament, NT; neurotransmitter, NTR; neurotransmitter receptor, PSD; postsynaptic density, SV; synaptic vesicle [file 40478_2022_1387_MOESM6_ESM.pdf]

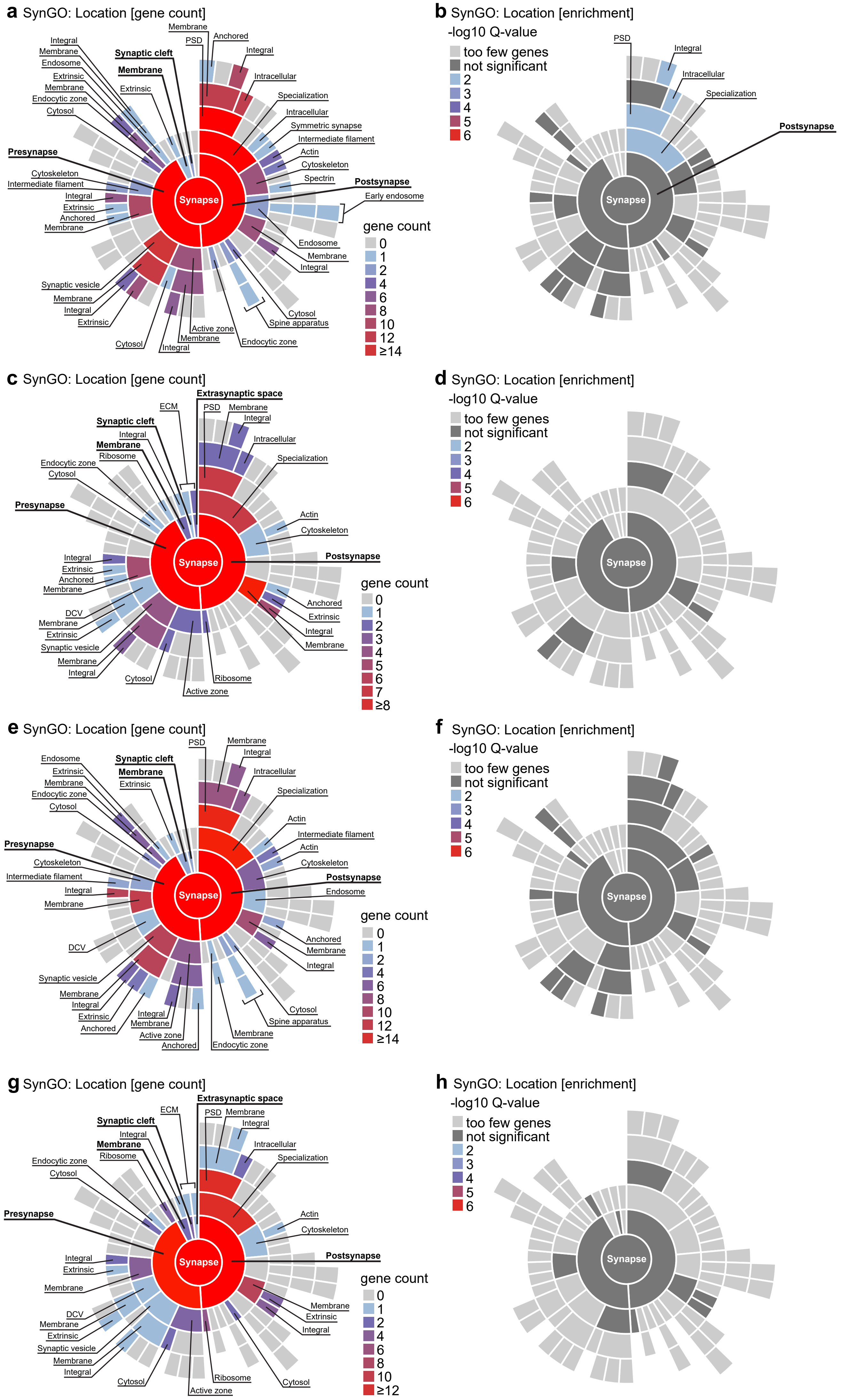
a SynGO: Location [gene count]
Membrane
PSD
Anchored
Integral
Membrane
Endosome
Synaptic cleft
Integral
Extrinsic
Membrane
Membrane
Intracellular
Endocytic zone
Extrinsic
Specialization
Cytosol
Intracellular
Symmetric synapse
Intermediate filament
Presynapse
Actin
Cytoskeleton
Cytoskeleton
Spectrin
Intermediate filament
Postsynapse
Integral
Early endosome
Extrinsic
Synapse
Anchored
Membrane
Endosome
Membrane
Integral
Synaptic vesicle
Membrane
Integral
Cytosol
Extrinsic
Spine apparatus
Active zone
Membrane
Endocytic zone
Cytosol
Integral
gene count
0
1
2
4
6
8
10
12
≥14
b SynGO: Location [enrichment]
-log10 Q-value
too few genes
not significant
2
3
4
5
6
PSD
Integral
Intracellular
Specialization
Postsynapse
Synapse
c SynGO: Location [gene count]
Extrasynaptic space
ECM
PSD
Membrane
Synaptic cleft
Integral
Integral
Intracellular
Membrane
Ribosome
Specialization
Endocytic zone
Cytosol
Presynapse
Actin
Cytoskeleton
Postsynapse
Integral
Extrinsic
Synapse
Anchored
Membrane
Anchored
DCV
Extrinsic
Membrane
Integral
Extrinsic
Membrane
Synaptic vesicle
Membrane
Integral
Cytosol
Ribosome
Active zone
gene count
0
1
2
3
4
5
6
7
≥8
d SynGO: Location [enrichment]
-log10 Q-value
too few genes
not significant
2
3
4
5
6
Synapse
e SynGO: Location [gene count]
PSD
Membrane
Endosome
Synaptic cleft
Integral
Extrinsic
Membrane
Intracellular
Membrane
Extrinsic
Endocytic zone
Specialization
Cytosol
Actin
Presynapse
Intermediate filament
Actin
Cytoskeleton
Cytoskeleton
Intermediate filament
Postsynapse
Integral
Endosome
Membrane
Synapse
Anchored
Membrane
DCV
Integral
Synaptic vesicle
Membrane
Integral
Extrinsic
Cytosol
Anchored
Integral
Spine apparatus
Membrane
Membrane
Active zone
Anchored
Endocytic zone
gene count
0
1
2
4
6
8
10
12
≥14
f SynGO: Location [enrichment]
-log10 Q-value
too few genes
not significant
2
3
4
5
6
Synapse
g SynGO: Location [gene count]
Extrasynaptic space
ECM
PSD
Membrane
Synaptic cleft
Integral
Integral
Membrane
Intracellular
Ribosome
Specialization
Endocytic zone
Cytosol
Presynapse
Actin
Cytoskeleton
Postsynapse
Integral
Extrinsic
Synapse
Membrane
Membrane
Extrinsic
DCV
Integral
Membrane
Extrinsic
Synaptic vesicle
Membrane
Integral
Cytosol
Ribosome
Cytosol
Active zone
gene count
0
1
2
4
6
8
10
≥12
h SynGO: Location [enrichment]
-log10 Q-value
too few genes
not significant
2
3
4
5
6
Synapse

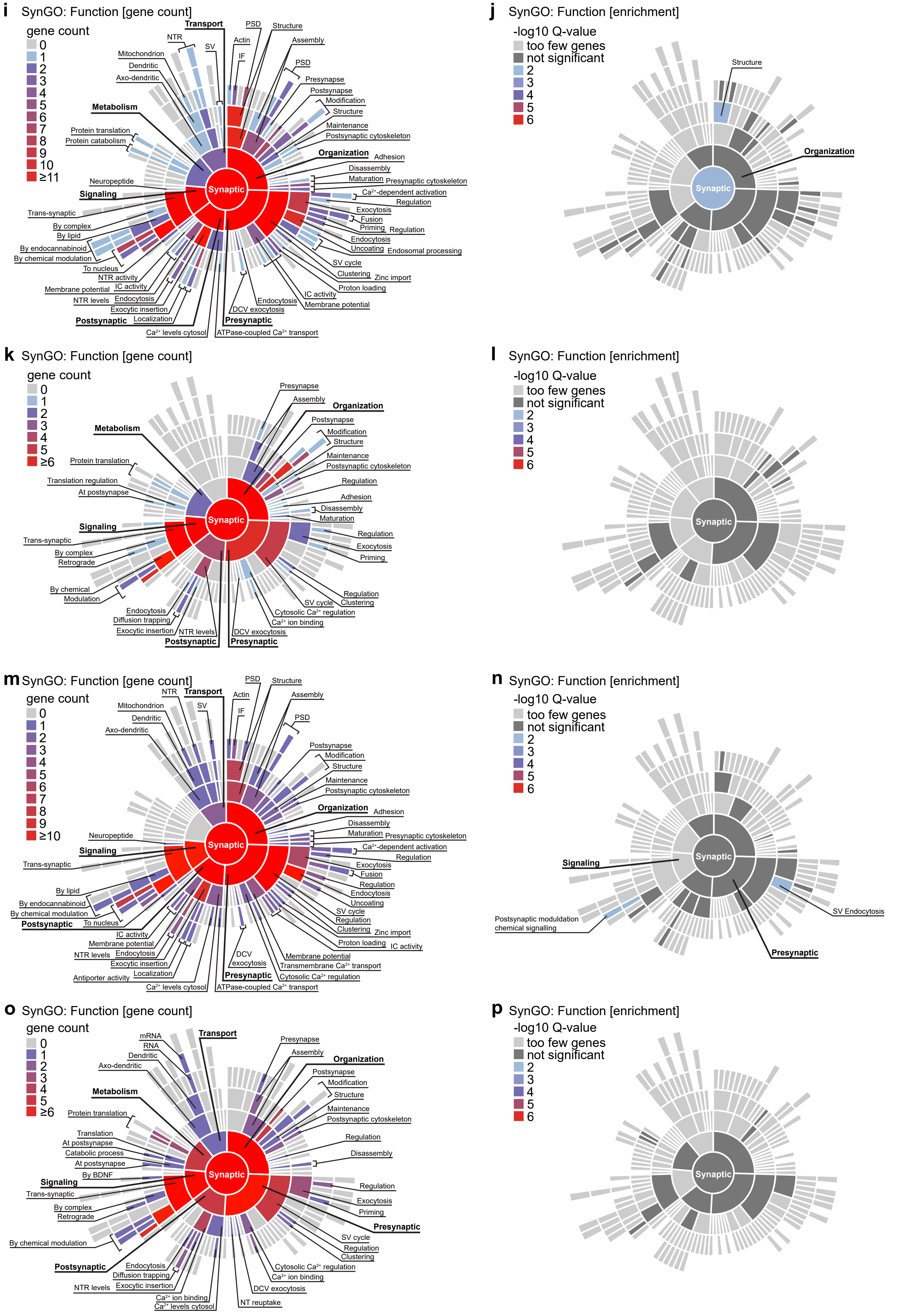

i
SynGO: Function [gene count]
gene count
0
1
2
3
4
5
6
7
8
9
10
≥11
Transport
NTR
SV
PSD
Structure
Actin
IF
Assembly
Mitochondrion
Dendritic
Axo-dendritic
PSD
Presynapse
Postsynapse
Modification
Structure
Maintenance
Postsynaptic cytoskeleton
Metabolism
Protein translation
Protein catabolism
Organization
Adhesion
Disassembly
Maturation
Presynaptic cytoskeleton
Neuropeptide
Signaling
Synaptic
Ca²⁺-dependent activation
Regulation
Trans-synaptic
Exocytosis
Fusion
Priming
Regulation
By complex
By lipid
By endocannabinoid
By chemical modulation
Endocytosis
Uncoating
Endosomal processing
To nucleus
NTR activity
SV cycle
Clustering
Zinc import
Membrane potential
IC activity
Proton loading
NTR levels
Endocytosis
Exocytic insertion
Endocytosis
IC activity
DCV exocytosis
Membrane potential
Postsynaptic
Localization
Presynaptic
Ca²⁺ levels cytosol
ATPase-coupled Ca²⁺ transport
j
SynGO: Function [enrichment]
-log10 Q-value
too few genes
not significant
2
3
4
5
6
Structure
Organization
Synaptic
k
SynGO: Function [gene count]
gene count
0
1
2
3
4
5
≥6
Presynapse
Assembly
Organization
Metabolism
Postsynapse
Modification
Structure
Maintenance
Postsynaptic cytoskeleton
Protein translation
Translation regulation
Regulation
At postsynapse
Adhesion
Disassembly
Maturation
Signaling
Synaptic
Regulation
Trans-synaptic
Exocytosis
By complex
Priming
Retrograde
By chemical
Modulation
Regulation
Clustering
Endocytosis
SV cycle
Diffusion trapping
Cytosolic Ca²⁺ regulation
Exocytic insertion
Ca²⁺ ion binding
NTR levels
DCV exocytosis
Postsynaptic
Presynaptic
l
SynGO: Function [enrichment]
-log10 Q-value
too few genes
not significant
2
3
4
5
6
Synaptic
m
SynGO: Function [gene count]
gene count
0
1
2
3
4
5
6
7
8
9
≥10
PSD
Structure
NTR
Transport
Actin
Assembly
Mitochondrion
SV
IF
Dendritic
PSD
Axo-dendritic
Postsynapse
Modification
Structure
Maintenance
Postsynaptic cytoskeleton
Organization
Adhesion
Disassembly
Neuropeptide
Maturation
Presynaptic cytoskeleton
Synaptic
Ca²⁺-dependent activation
Signaling
Regulation
Trans-synaptic
Exocytosis
Fusion
Regulation
By lipid
Endocytosis
By endocannabinoid
Uncoating
By chemical modulation
SV cycle
Regulation
Postsynaptic
To nucleus
Clustering
Zinc import
IC activity
Proton loading
IC activity
Membrane potential
NTR levels
Endocytosis
DCV
exocytosis
Membrane potential
Exocytic insertion
Transmembrane Ca²⁺ transport
Presynaptic
Cytosolic Ca²⁺ regulation
Antiporter activity
Localization
Ca²⁺ levels cytosol
ATPase-coupled Ca²⁺ transport
n
SynGO: Function [enrichment]
-log10 Q-value
too few genes
not significant
2
3
4
5
6
Signaling
Synaptic
Postsynaptic moduldation
chemical signalling
SV Endocytosis
Presynaptic
o
SynGO: Function [gene count]
gene count
0
1
2
3
4
5
≥6
mRNA
Transport
RNA
Presynapse
Dendritic
Assembly
Axo-dendritic
Organization
Metabolism
Postsynapse
Modification
Structure
Maintenance
Protein translation
Postsynaptic cytoskeleton
Translation
Regulation
At postsynapse
Catabolic process
Disassembly
At postsynapse
By BDNF
Synaptic
Signaling
Regulation
Trans-synaptic
Exocytosis
By complex
Priming
Retrograde
Presynaptic
SV cycle
By chemical modulation
Regulation
Clustering
Postsynaptic
Endocytosis
Cytosolic Ca²⁺ regulation
Diffusion trapping
Ca²⁺ ion binding
NTR levels
Exocytic insertion
DCV exocytosis
Ca²⁺ ion binding
Ca²⁺ levels cytosol
NT reuptake
p
SynGO: Function [enrichment]
-log10 Q-value
too few genes
not significant
2
3
4
5
6
Synaptic
